# Vesicles, fibres, films and crystals: A low-molecular-weight-gelator [Au(6-thioguanosine)_2_]Cl which exhibits a co-operative anion-induced transition from vesicles to a fibrous metallo-hydrogel†

**DOI:** 10.1039/d3sm01006f

**Published:** 2023-10-19

**Authors:** Liam F. McGarry, Osama El-Zubir, Paul G. Waddell, Fabio Cucinotta, Andrew Houlton, Benjamin R. Horrocks

**Affiliations:** a Chemistry, School of Natural Sciences, Newcastle University, Newcastle upon Tyne NE1 7RU UK ben.horrocks@ncl.ac.uk

## Abstract

We describe a simple coordination compound of Au(i) and 6-thioguanosine, [Au(6-tGH)_2_]Cl, that has a rich self-assembly chemistry. In aqueous solution, the discrete complex assembles into a supramolecular fibre and forms a luminescent hydrogel at concentrations above about 1 mM. Below this concentration, the macromolecular structure is a vesicle. Through appropriate control of the solvent polarity, the gel can be turned into a lamellar film or crystallised. The molecular structure of [Au(6-tGH)_2_]Cl was determined using single crystal X-ray diffraction, which showed bis-6-thioguanosine linearly coordinated through the thione moiety to a central Au(i) ion. In the vesicles, the photoluminescence spectrum shows a broad, weak band at 550 nm owing to aurophilic interactions. Co-operative self-assembly from vesicle to fibre is made possible through halogen hydrogen bonding interactions and the aurophilic interactions are lost, resulting in a strong photoluminescence band at 490 nm with vibronic structure typical of an intraligand transition. The vesicle-fibre transition is also revealed by a large increase of ellipticity in the circular dichroism spectrum with a prominent peak near 390 nm owing to the helical structure of the fibres. Atomic force microscopy shows that at the same time as fibres form, the sample gels. Imaging near the vesicle-fibre transition shows that the fibres form between vesicles and a mechanism for the transition based on vesicle collisions is proposed.

## Abstract 2

[Au(6-thioguanosine)_2_]Cl has a rich self-assembly chemistry which is unravelled using the response of its intrinsic luminescence to intermolecular interactions.

## Graphical abstract

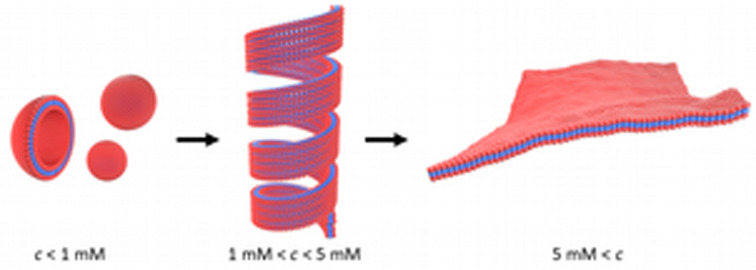

## Introduction

Self-assembly in aqueous solution is a fundamental feature of biology, along with the associated stimuli-responsive dynamical change between the range of resulting morphologies, which cover vesicles/micelles, fibres, rods/tubes and sheets.^1,2^ The ability to design synthetic systems, especially of low molecular weight, that display such behavior can provide useful insight into (pre-)biological processes and is important for developing new materials.^3,4^ However, this remains a considerable challenge.

A major issue is that in water the ability to exploit directional hydrogen-bonding in the self-assembly, as is common in organic solvents, is reduced owing to competition from the solvent. However, an increasing understanding of the principal factors, both qualitatively and quantitatively, has allowed progress to be made. In addition to hydrogen-bonding, these factors include the hydrophobic effect, and it is now considered that hydrophobic surfaces must exceed *ca.* 1 nm^2^ to assist aggregation.^5–7^ Examples of small-molecule self-assembly in aqueous solution, which exploit this understanding, have been developed, although these are overwhelmingly organic systems.^3,8–10^

Coordinate bond formation is another useful tool for supramolecular design^11,12^ and in aqueous solution, with judicious choice of metal/ligand combination, the competitive metal-binding of water can be minimised. Furthermore, for any coordination number >1, metal–ligand bond formation provides a synthetically straightforward means to increase both the number of hydrogen bonding groups and to increase the surface area of hydrophobic ligand moieties. Finally, for certain metals, particularly silver^13^ and gold,^14^ metallophilic interactions are available to assist in intermolecular assembly.

In an effort to exploit the range of features afforded by metal-ions to design a minimal system capable of aqueous self-assembly we noted that the earliest life forms, archaebacteria, use bolaamphiphiles to assemble their uniquely simple monolayered membrane.^15^ Bolaamphiphiles, in which two hydrophilic headgroups are connected by a hydrophobic region, show a variety of self-assembled structures though these have principally been organic systems.^16–19^ We reasoned that Au(i), which commonly adopts linear coordination geometry, is well suited to provide this type of molecular arrangement and, furthermore, as it can exhibit metallophilic interactions make it an ideal candidate. Nucleosides provide a promising combination of well-separated hydrophilic/hydrophobic groups and are versatile ligands suited for self-assembly in aqueous media and the formation of hydrogels.^20^ Whilst none of the natural nucleosides are well-adapted for binding soft ions, the thio-derivatives,^21^ where S replaces O on the nucleobase, as in 6-thioguanosine, are.^22–24^

Using these design principles, we report a low molecular weight gelator (LMWG) and its aqueous self-assembly in the form of a two-coordinate cationic complex gold(i)-bis(-)-6-thioguanosine, [Au(i)-((-)6-tGH)_2_]^+^ which displays an array of different morphologies. Driven initially by metal–ligand bond formation, subsequent hydrophobic effects, aurophilic interactions and hydrogen bonding this complex assembles into vesicles, then fibres, and then lamellae with the process responding to concentration, temperature and chloride ions.

## Results and discussion

This manuscript is organised as follows: first, we discuss the evidence for the different morphologies and then the characteristics of the transitions between these states (vesicle-fibre and fibre-lamellae) are described. We start with the molecular structure obtained from single crystal diffraction. Next, the evidence for the vesicles, the helical fibres and the lamellae is presented. The vesicle-fibre transition and its relation to gelation is discussed using atomic force microscopy to provide evidence for a mechanism based on collision between vesicles. The vesicle-fibre and fibre-lamellae transitions also result in profound changes in the optical spectroscopy (photoluminescence and circular dichroism). This allows a demonstration of the cooperative nature of the vesicle-fibre transition and the important role of chloride ion hydrogen bonding interactions.

### [Au(6-tGH)_2_]Cl, 1, as a discrete complex

Compound 1, as colourless crystals, was isolated from the aqueous reaction of ribonucleoside (−)6-thioguanosine (6-tGH) and Au(i) ions in a 2 : 1 ratio after vapour diffusion of acetone and shown to be [Au(S6-6-tGH)_2_]Cl·3H_2_O by single crystal X-ray crystallography. The molecular cation was also observed in solution by ES-MS as [Au(6-tGH)_2_]^+^ with the dominant isotope peak at *m*/*z* = 795.1042 amu (ESI,† Fig. S1). Powder X-ray diffraction of the sample was recorded to provide evidence of purity; the experimental pattern matched that computed from the single crystal data (ESI,† Fig. S2).

**Fig. 1:**
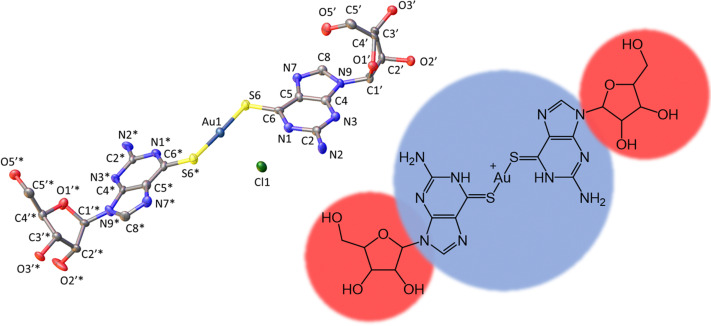
Molecular structure of the cation [Au(6-tGH)_2_]^+^ (1) with ellipsoids drawn at the 50% probability level. Left: Au(i) = dark blue; Cl^−^ = green; S = yellow; O = red; N = light blue and C = grey. Right: Hydrophobic (blue) metal-nucleobase region and the hydrophilic (red) sugar groups.

**Fig. 2:**
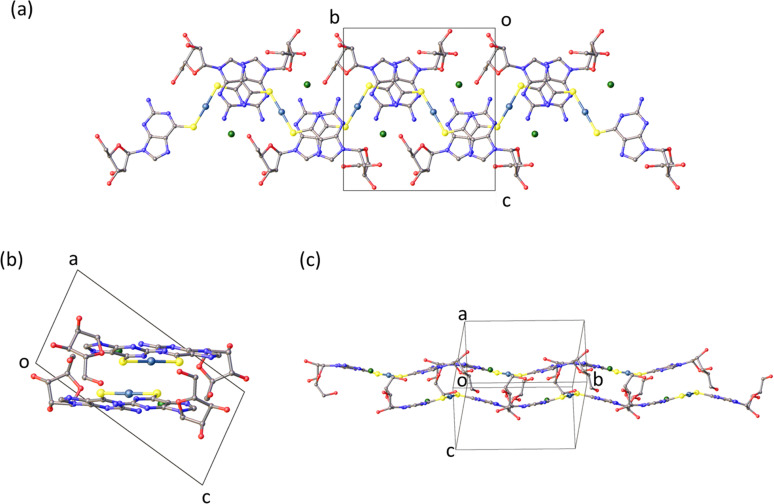
Olex2 images of [Au(6-tGH)_2_]Cl chains found in the crystal structure (a) plan view of the chains. (b) View showing the base-stacking. (c) Along the side of the chains.

Fig. 1 shows the molecular structure of the cation which features linear coordination geometry at the Au(i) centre and two neutral 6-thioguanosine ligands bound *via* sulfur to the metal ion. The Au–S bonds are 2.2741(1) and 2.2810(1) Å and the S–Au–S bond angle is 178.25(1)°. The two nucleobases are twisted slightly out of co-planarity by 13.5(1)°. The carbon–sulfur distances for each ligand are 1.701(1) and 1.726(1) Å, which is indicative of the thione, rather than the thiol, form. The thiol form has a longer average C–S bond length of about 1.77 Å compared to typical thione C=S bond lengths which are about 1.72 Å. [ESI,† section 8]. The nucleoside conformations are intermediate between *syn*/*anti* because the C8–H8 bond of the purines are aligned with the C1′–C2′ bond of the ribose unit (angles of 1.6/9.6° between planes defined by N7–C8–H8 and N9–C1′–C2′) and these adopt C3′-*endo* and C3′-*exo* arrangements, respectively. In addition to this cation, the crystal lattice contains a chloride anion, which balances the positive charge of the molecular complex, and three water molecules, all of which are involved in multiple hydrogen bonding interactions with the complex cations (Fig. 2).

The total surface area and molecular volume of 1 are 5.46 nm^2^ and 0.447 nm^3^. In Fig. 1 the sugar groups are coloured red indicating that these are hydrophilic, and the nucleobase/metal regions are coloured blue to denote hydrophobic regions of the molecule. In schematics which do not show atomic detail, we retain this colouring scheme to show how 1 self-assembles in aqueous solution. It is useful to estimate the hydrophobic surface area (3.44 nm^2^) because this is an important factor in determining capability for self-assembly.^5–7^

### Crystalline solid

The molecular packing in the solid (Fig. 2) features bilayer chains of [Au(6-tGH)_2_]^+^, and associated chloride ions and water molecules, with the purine moieties internalised and the ribose presented at the edge as viewed in Fig. 2a. An individual bilayer is *ca.* 14.5 Å wide and 3.76 Å thick, as defined by the shortest distances between appropriate O3′⋯O3′ and N7⋯N7 atoms, respectively. The inter-bilayer spacing is 14.84(1) Å measured between corresponding Au(i) ions in neighbouring layers (ESI,† Fig. S9). A combination of hydrogen bonds and (slipped) base stacking interactions assemble these bilayers. The closest inter-metallic distance is 7.332(1) Å indicating a lack of aurophilic interactions in the solid-state. Within a single bilayer, two bases form hydrogen bonds between N2H and N3 (N2⋯N3*, 3.054(16) Å; N2*⋯N3, 3.028(17) Å). One layer of a bilayer chain is connected to the other *via* hydrogen bonds from the sugar group of one complex to the base of another in the second layer with the OH groups acting as donor in the first case and acceptor in the others (O5′⋯N7*, 2.788(14) Å; O5′*⋯N1, 2.818(13) Å; O5′*⋯N2, 2.875(16) Å). The chloride ion exhibits four hydrogen bonding interactions. Three of these interactions are within a single layer with protons attached to N1, N2, and O5′ (Cl1⋯N1, 3.182(10) Å; Cl1⋯N2, 3.258(12) Å; Cl1⋯O5′*, 3.081(11) Å), and the fourth is from the O2’-bound proton from the adjacent layer (Cl1⋯O2′, 3.157(12) Å). The latter is discussed further in the context of fibre formation and displayed in Fig. 15.

### Self-assembly of [Au(6-tGH)_2_]^+^

**Scheme 1:**
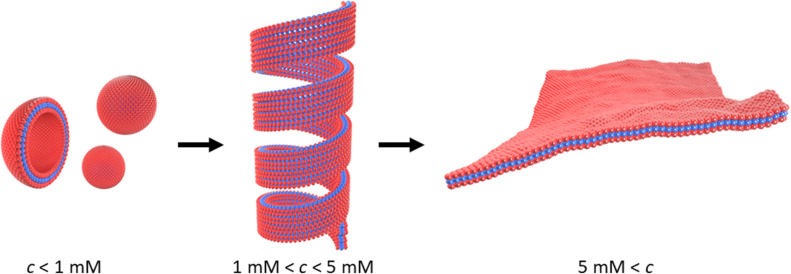
Transformation of vesicles (left) to long fibres in the form of helical ribbons (centre) at the minimum gelation concentration (c*) of approximately 1 mM. At higher concentrations, *c* > 5 mM, or in the presence of antisolvent, the helical ribbons unwind and lamellae form (right). The red colour indicates the sugar moiety (hydrophilic), and the blue colour indicates the Au(i)-thionucleobase moiety (hydrophobic) as shown in Fig. 1.

Scheme 1 outlines the observed self-assembly phenomenology of [Au(6-tGH)_2_]^+^ (1) as a function of concentration. The hydrophilic sugar moieties (red) on either side of the hydrophobic Au(i)-thionucleobase (blue) constitute a bolaamphiphile. At concentrations below the minimum gelation concentration c* ≈ 1 mM at 20 °C, vesicles are present. At c* an abrupt transition from vesicles to fibres occurs and a hydrogel forms. We refer to these linear structures as fibres for convenience; their precise form is that of a helical ribbon. This transition is clearly observed by electron and probe microscopy. Alongside the vesicle-fibrous gel transition, marked changes also occur in the electronic spectra, especially the photoluminescence and circular dichroism spectra. At higher concentrations, about *c* = 5 mM, the system remains a hydrogel, but some of the fibres unwind to produce lamellae. Upon appropriate treatment with antisolvents or by freeze-drying, these lamellae can be obtained as single layer films on a substrate suitable for characterisation by atomic force microscopy (AFM). Finally, slow, vapour-diffusion of antisolvent into the gel can be employed to obtain crystals suitable for structure solution by X-ray diffraction.

### Fibres and vesicles: electron microscopy and *in situ* atomic force microscopy

**Fig. 3:**
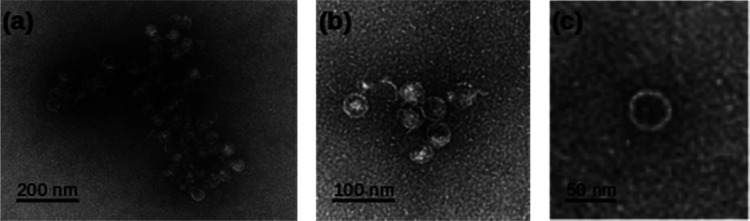
TEM images of 0.5 mM [Au(6-tGH)_2_]Cl employing uranyl acetate as a negative stain. (a), (b) Aggregates of vesicles and (c) a single vesicle at higher resolution.

Direct evidence for the formation of vesicles was obtained by transmission electron microscopy (TEM) on samples prepared at *c* = 0.5 mM. A negative stain (uranyl acetate) was employed to increase the image contrast in Fig. 3. The micrographs show circular features consistent with spherical vesicles, although there is evidence of some damage and aggregation which may have occurred during the staining and drying process. The vesicles are notably monodisperse and we estimated a mean diameter of 40.4 ± 1.1 nm based on a sample of 20 vesicles.

**Fig. 4:**
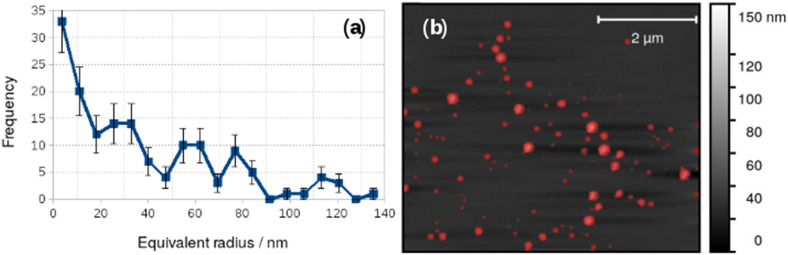
Particle size analysis of vesicles in 0.2 mM [Au(6-tGH)_2_]Cl in aqueous solution. (a) Histogram of the equivalent radii of the objects detected by the threshold mask (red colour) in the AFM image; (b) *in situ* AFM image of vesicles on a Si(100) substrate.

Vesicles were also observed with AFM and Fig. 4 shows an image acquired in liquid in a 0.2 mM aqueous solution of 1 on a Si(100) substrate. A height threshold mask has been applied to detect particles (Fig. 4b) for analysis. The particle analysis determined the radius of a disc with the same area as each object detected. The corresponding distribution of equivalent radii is shown in Fig. 4a. The mean diameter of the vesicles observed by *in situ* AFM in Fig. 4 was 73 nm. It can be clearly seen that the diameter distribution of the vesicles in Fig. 4 is polydisperse and that there are many vesicles much larger than observed in the dry state by electron microscopy (Fig. 3.). Vesicles of alkynyl Au(i) complexes ([Au(C≡C–C_5_H_4_N–CH_3_)(PTA)]I) have been observed and in these samples the smallest diameters were about 20 nm but also multilayer systems up to 130–140 nm were found at higher concentration.^25^

**Fig. 5:**
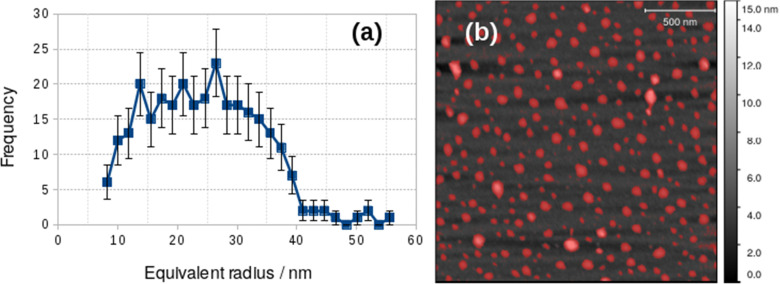
Particle size analysis of vesicles dried onto a Si(100) substrate from 0.2 mM [Au(6-tGH)_2_]Cl aqueous solution. (a) Histogram of the equivalent radii of the objects detected by the threshold mask (red colour) in the AFM image; (b) AFM image of vesicles on a Si(100) substrate.

The difference between the TEM and *in situ* AFM measurements likely reflects a combination of vesicle–substrate interactions in AFM and the effect of drying during sample preparation for the TEM measurements. Further support for the effect of drying on the vesicle size distribution was obtained from AFM images obtained on samples dried in air onto Si(100) chips. These images showed a mean diameter of 48.8 ± 19 nm, similar to that obtained from TEM, but with a broader distribution (Fig. 5).

**Fig. 6:**
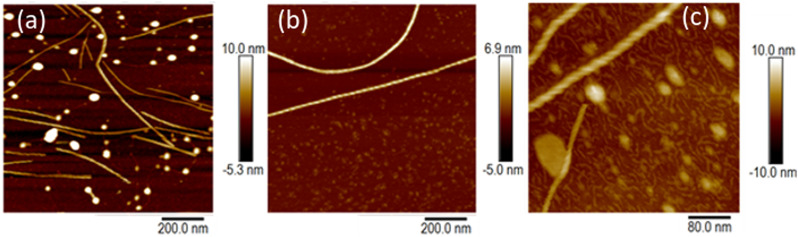
AFM images of [Au(6-tGH)_2_]Cl on silicon (100) chips. (a) *in situ* AFM in aqueous solution at a concentration of *c* = 1 mM close to the minimum gelation concentration. (b) *Ex situ* AFM in air of a sample prepared by slow drying from a solution of *c* = 60 μM concentration. (c) *In situ* AFM at concentration *c* = 1 mM with 20 equivalents of urea added.

At higher concentrations we observed fibres (Fig. 6) and the sample gels. Fig. 6a shows an *in situ* AFM image at *c* = 1 mM in which both vesicles (bright spots) and helical fibres (the linear structures) are observed. These fibres are clearly thicker than a simple monomolecular chain of 1 and show a periodicity and a twist in the images (Fig. 6b and c). They may be described as helical ribbons as in Scheme 1. Vesicle-fibre transitions are of substantial interest because they appear to be a general phenomenon in a range of molecular systems.^26^ They are well-known in organic systems,^26–30^ but metallo-supramolecular systems are less common. However, an Au(i) complex has been prepared that assembles vesicles and fibres in THF/water mixtures.^31^

Fig. 6b shows an AFM image of a fibre formed by slow drying of a dilute solution (*c* = 60 μM) onto a Si(100) substrate. As the water evaporates, the concentration of 1 increases and fibres can be observed. In the example shown, the right-handed helical nature of the fibre running through the centre of the image is clear. The fibres in Fig. 6a and b appear to be stiff, with a radius of curvature greater than 100 nm and are more likely to be ribbons as illustrated in Scheme 1 than individual chains. Further, the diameter of the features in Fig. 6a is 6.4 ± 1.0 nm (based on profiles of 12 fibres). This is too large to be a single chain of monomers of [Au(6-tGH)_2_]^+^. From sugar to sugar (3′ O atoms) the diameter of the monomer (1) is about 2.2 nm; therefore, we suggest that the helical fibres observed are in the form of a twisted, helical ribbon as illustrated in Scheme 1. Further support for this interpretation was obtained by *in situ* imaging of the helical ribbons after the introduction of the chaotropic denaturant, urea. Fig. 6c shows a high-resolution image under these conditions. Two helical ribbons are observed in the top left of the image; however, urea causes many of the ribbons to disaggregate and the image shows much thinner structures with a diameter of about 2 nm which can be assigned to short fibres of monomolecular diameter.

### Lamellae and crystals: electron microscopy, X-ray diffraction and atomic force microscopy

At higher concentrations, *c* > 5 mM, the helical structures observed in Fig. 6 unwind and lamellae are formed. The xerogel obtained by freeze-drying a gel at 6 mM is dominated by sheet-like structures (ESI,† Fig. S4). Alternately, it is possible to produce lamellae by addition of an antisolvent such as acetone and even to crystallise 1 by vapour in-diffusion. This is of interest and was investigated by AFM and XRD because supramolecular gels which show gel-crystal transitions are considered uncommon; many systems either remain as gels or crystallise first.^32^

**Fig. 7:**
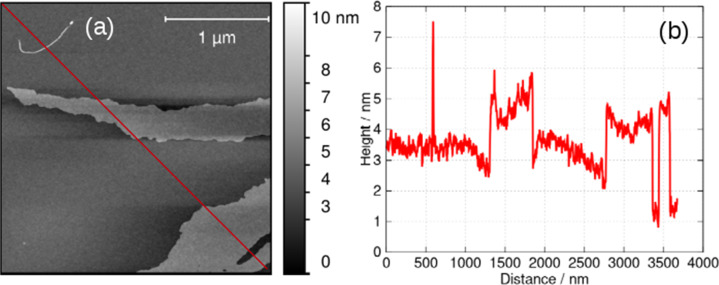
(a) AFM image of a fragment of a lamella of 1 formed by addition of an equal volume of acetone to a 2 mM aqueous gel, drop-casting onto an Si(100) wafer and drying. (b) Height profile along the red line shown in (a).

The lamellae were observed by electron microscopy (ESI,† Fig. S5) and AFM. Fig. 7 shows an AFM image of a fragment of a monomolecular layer formed after addition of 50% by volume acetone to a 2 mM aqueous gel of 1 and drop-casting onto a Si substrate. The height profile in Fig. 7b crosses a fibre visible at the top left of Fig. 7a and two lamellae in the centre and the bottom right of the image. The fibre has a diameter of about 4.5 nm, whereas the lamellae have a thickness of about 2 nm which is consistent with the diagram of Scheme 1, the diameter of the single strand fibres of Fig. 6c and the crystallographic data for the sugar–sugar distance based on the 3′ O atoms.

**Fig. 8:**
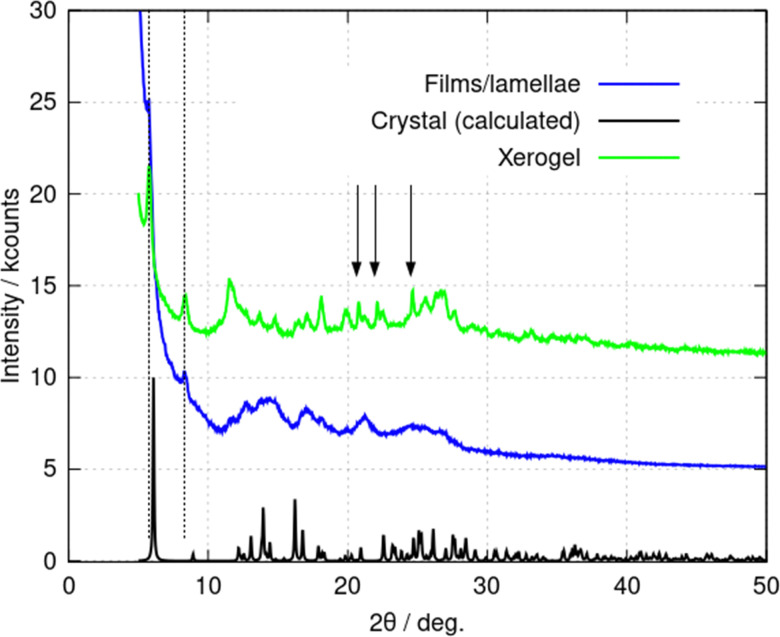
Powder X-ray diffraction (PXRD) patterns of (1) in various forms. The freeze-dried xerogel, *c* = 10 mM (green line); the film formed through rapid addition of acetone to a *c* = 2 mM gel (blue line); and the predicted PXRD pattern from the crystal structure formed upon vapour diffusion of acetone (black line). The blue and green traces are offset by 5 kcounts and 10 kcounts for clarity. The vertical arrows indicate peaks assigned to 2,2′-sulfinyldiethanol. The dotted lines indicate the peaks discussed in the text.

Additional information on the lamellae was obtained from powder X-ray diffraction (PXRD) data. Three types of samples were prepared: (i) a freeze-dried xerogel; (ii) films produced by rapid addition of acetone and (iii) crystals produced by vapour-diffusion of acetone. The xerogel shows a broad, indistinct scattering pattern, however, sharper peaks appear gradually upon vacuum drying (ESI,† Fig. S6a). Fig. 8 compares PXRD patterns for each of these samples; the freeze-dried xerogel in this figure was dried under vacuum for two weeks. Three peaks assigned to the oxidised form (2,2′-sulfinyldiethanol) of the reducing agent used to produce Au(i) are marked by black arrows. The PXRD pattern of 2,2′-sulfinyldiethanol is shown in (ESI,† Fig. S6b) for reference. The freeze-dried xerogel and the films both show peaks at 2*θ* = 5.79° and 8.42° (Fig. 8. dotted lines). The pattern obtained from the films has much broader peaks. The calculated PXRD pattern for 1 in Fig. 8 was computed from the single crystal diffraction data. This shows an (001) reflection at 2*θ* = 6.08° and an (011) reflection at 2*θ* = 8.96°. These features are like those in the freeze-dried xerogel and the films, but with small decreases in the interplanar distances upon crystallisation. PXRD data for crystals formed by vapour-diffusion of isopropanol also showed these features, although with some changes to peak intensity, which may represent effects of inclusion of isopropanol in the structure (ESI,† Fig. S7).

AFM was used to investigate the crystallisation. A vapour diffusion experiment was set up with acetone diffusing into the gel in the presence of an Si chip (ESI,† Fig. S10). Once crystals started to form, the Si chip was removed and dried. AFM was used to analyse the crystal formation on the Si substrate (ESI,† Fig. S11). The AFM images show the growth of needle-shaped crystals from the network of fibres in the gel. We suggest that the crystals (ESI,† Fig. S11a bottom and Fig. S11b top left) are more rigid than the fibres because they do not show evidence of bending. Optical micrographs of the crystals are also shown in the (ESI,† Fig. S12). Under the optical microscope, the large crystals take the form of needles with a length approaching 50 μm and a width of about 5 μm.

### Gelation and the vesicle-fibre transition

Vesicle to fibre transitions in LWMG are known in amino acids, short peptides and organic molecules.^27^ The switching process is often controlled by changes in concentration, pH, temperature, light, and other environmental stimuli, including the addition of metal (Ag(i)) ions.^33^ This sort of hierarchical self-assembly has been seen in some organometallic systems and properties, such as luminescence, arising from the presence of the metal ion, facilitate study of the self-assembly.^31^

A supramolecular hydrogel forms when aqueous solutions of 1 exceed a minimum gelation concentration of about 1 mM at 20 °C. Gelation was observed to be sensitive to temperature, concentration, and anion identity. The typical time for a 1 mM solution to gel was of the order of 18 h, but this decreased with increasing concentration. For example, a 10 mM solution would typically gel in a time of 1–2 h.

A crude indication of the formation of a hydrogel is provided by the inversion test (ESI,† Fig. S13). The formation of a hydrogel was confirmed by rheology using frequency sweep measurements to determine the mechanical properties (ESI,† Fig. S14). The storage modulus value (*G*′) is larger than the loss modulus (*G*′′) over the range of frequencies from 0.1 to 100 rad s^−1^ (ESI,† Fig. S14b). These measurements indicate that the sample behaves like a viscoelastic solid rather than a viscous liquid in this parameter range. An amplitude sweep shows a linear-viscoelastic region up to about 5 μN m (ESI,† Fig. S14c) and a flow sweep test shows that the solution is thixotropic with a decrease in viscosity as shear rate increases (ESI,† Fig. S14d). This behaviour is typical of a supramolecular gel.^34^

Supramolecular systems also respond readily to temperature and therefore we investigated the temperature-dependence of properties of 1 in order to distinguish supramolecular gelation from gelation by formation of a coordination polymer as in the case of poly(Au6tG).^24^ We prepared a 4.5 mM sample in D_2_O and recorded ^1^H NMR spectra over the temperature range 298–363 K (ESI,† Fig. S15). Broad and indistinct peaks, typical of a gel, were seen at 298 K, but as the temperature was increased to 363 K these peaks sharpened and the ^1^H NMR spectrum can be assigned to that expected for monomeric [Au(6-tGH)_2_]^+^ (ESI,† Fig. S16–S20). The interpretation is that the supramolecular structure is dismantled at higher temperature, leaving single molecules, and increasing the tumbling rate to produce high-resolution spectra; this is also a characteristic feature of supramolecular systems.^35^

### Mechanism of the vesicle-fibre transition

**Scheme 2:**
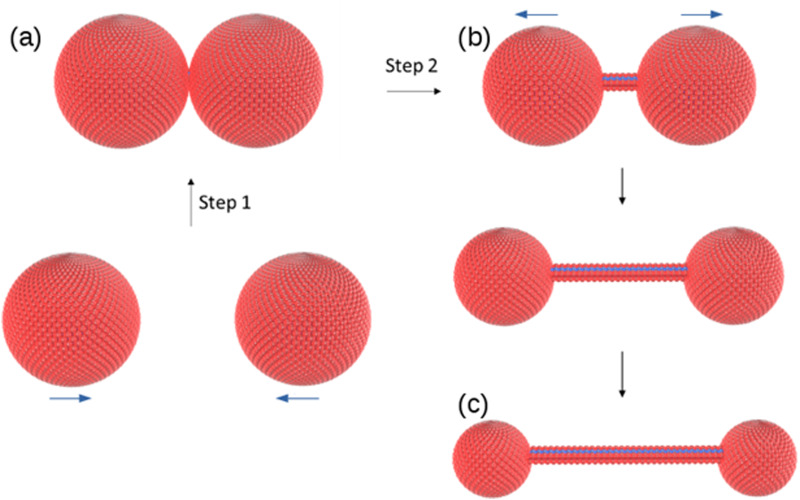
Suggested mechanism for the vesicle-fibre transition. (a) two vesicles collide; (b) a single molecular strand with diameter comparable to the molecule 1 forms between vesicles and (c) the strand lengthens as the vesicles separate and diminish in size.

The change in morphology of the self-assembled structures at the vesicle-fibre transition is striking and it is not obvious how this can occur. We do not see, for example, a gradual elongation or coalescence of the vesicles.^31^ However, a study of *in situ* AFM images near the transition provides evidence for a novel mechanism involving vesicle collisions illustrated in Scheme 2.

**Fig. 9:**
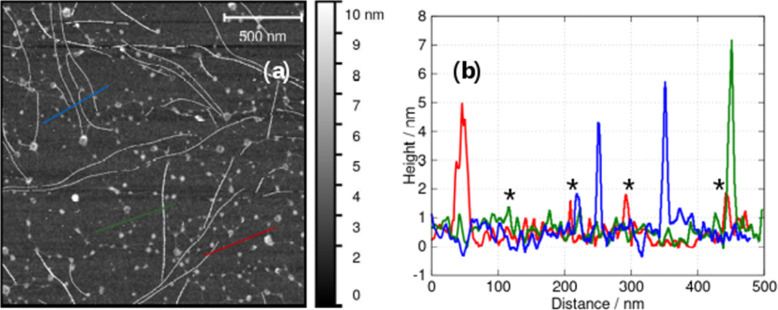
(a) *In situ* AFM image at the minimum gelation concentration of about 1 mM and (b) height profiles along the blue, green and red lines in (a). Examples of fibres whose AFM heights are compatible with single molecular chains are marked with an asterisk.

In AFM images taken under liquid near the minimum gelation concentration at *c* = 1 mM both vesicles and fibres are present (Fig. 9). Below the minimum gelation concentration only vesicles were observed as shown in Fig. 5 and 6. Above 1 mM, the vesicles transform into fibres, however this does not occur by an elongation of the vesicles. Instead, Fig. 9b shows both thick fibre bundles of height 5–7 nm and much thinner structures (strands) of height 1–2 nm which are consistent with the dimensions of the molecule (Fig. 1.). Careful examination of the image in Fig. 9a shows that all these thin strands connect vesicles (the bright spots) and that such strands do not occur on their own. Additional images are given in the (ESI,† Fig. S3).

The observations of Fig. 9 suggest a mechanism for initiating the vesicle-fibre transition shown in Scheme 2. A pair of vesicles comes into contact and then a strand forms between the two vesicles in a manner reminiscent of the pili of bacterial conjugation.^36^ The requirement for contact between two vesicles gives a rationalisation of the concentration-dependent nature of the transition.

**Fig. 10:**
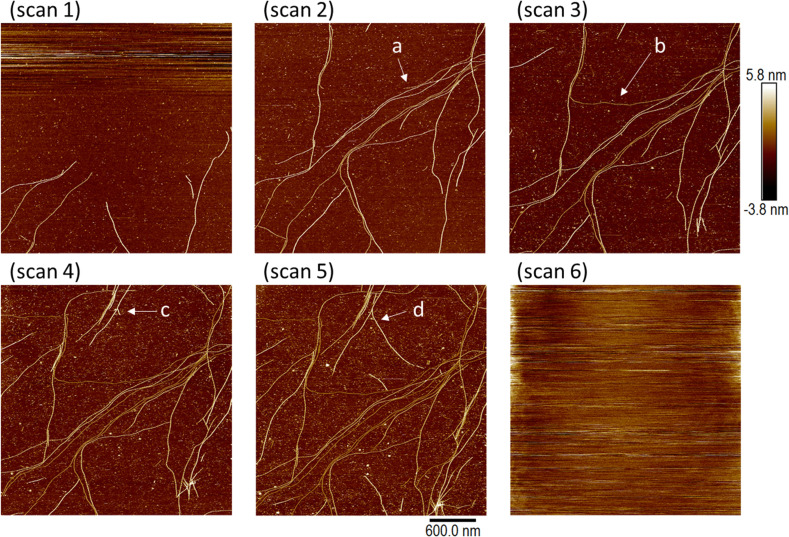
A set of *in situ* AFM images showing the self-assembly of fibres of 1 on a Si surface in an aqueous solution. A 3 mM sample of 1 was diluted to 1 mM and then a series of 6 images of a Si (100) chip was acquired in sequence; scan 1 to scan 6. All images have the same colour scale (−3.8 to + 5.8 nm, black to white) and the same image size (the scale bar indicates 600 nm). Each image required 17 min to acquire, and the subsequent image scan commenced immediately after the previous image was complete. The extension of individual fibres is clearly seen by comparison of the two images at points *a* and *b*, and comparison of points *c* and *d*. The viscosity of the sample greatly increased between scan 5 and scan 6 and image resolution was lost at this time.

The subsequent formation of thick fibres and gelation can be followed by *in situ* AFM near the minimum gelation concentration. In view of the sensitivity of the gelation time to the concentration, we proceeded by diluting a gel to a point near the minimum gelation concentration in order to study a case compatible with typical AFM frame acquisition times. Fig. 10 shows a sample of 3 mM [Au(6-tGH)_2_]Cl that was diluted to 1 mM and then analysed by *in situ* AFM on a (100)-oriented silicon chip as substrate. Scans 1–6 are images of the same region of the substrate taken in chronological order, about 17 minutes apart with no gaps between completion of the acquisition of one frame and the start of the next. In scan 1 a small number of comparatively short fibres is observed at the bottom of the imaged area, but in scan 2 the fibres span the width of the image. Extension of individual fibres can be clearly observed by comparing point (a) in scan 2 to point (b) in scan 3, and point (c) in scan 4 to point (d) in scan 5. The sequence of images shows the extension of individual fibres that happen to be adsorbed on the Si substrate during the gelation process. The density of coverage increases from scan 1 to scan 5 and after this imaging became impossible because of the increase of viscosity caused by incipient formation of a fibrous gel in the solution (scan 6).

### Effect of the vesicle-fibre and fibre-lamellae transitions on the optical spectroscopy

The presence of the Au(i) ion and the chirality of 6-thioguanosine give rise to structure-dependent changes in the spectroscopic and photophysical properties of 1. In this section we describe the relevant features of the optical spectroscopy of 1 which are useful for studying the vesicle-fibre and fibre-lamellae transitions that are observed (Scheme 1). In particular, the intermolecular aurophilic interactions and the circular dichroism exhibited by the sample are sensitive to the self-assembled structures.

**Fig. 11:**
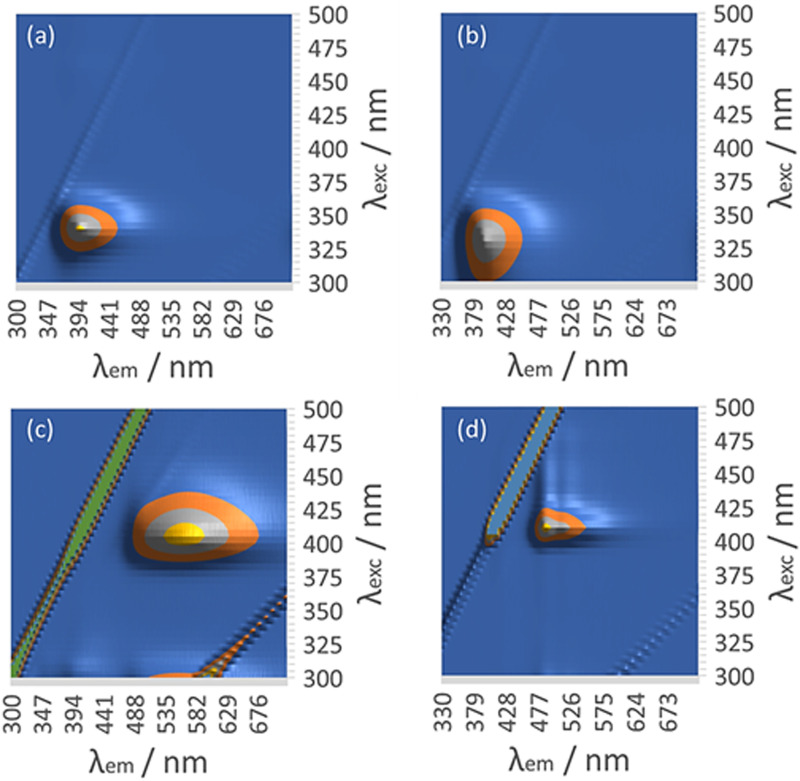
Luminescence excitation–emission matrix (EEM) of (a) 6-tGH at 60 μM. (b) [Au(6-tGH)_2_]Cl at 60 μM. (c) [Au(6-tGH)_2_]Cl at 0.5 mM. (d) [Au(6-tGH)_2_]Cl at 3 mM.

The absorption spectrum of the ligand 6-thioguanosine has a *λ*_max_ at 344 nm (ESI,† Fig. S21). The excitations are localised on the thione group and assigned to n_S_ → π* and π → π* transitions.^37^ The (n_S_ → π*) transition to the S_1_ state has a lower oscillator strength than the (π → π*) transition to S_2_. Excitation of 6-thioguanosine at *λ*_exc_ = 340 nm will therefore mainly populate the S_2_ state.^37^ The photoluminescence (PL) spectrum of 6-thioguanosine shows an unstructured peak with *λ*_em_ at 390 nm in the excitation–emission matrix (EEM) shown in Fig. 11a. Vibronic structure has been observed in low temperature glasses at 77 K in water and ethanol but at a longer wavelength.^38^ PL excitation and emission spectra of monomeric 1 at 5 μM (ESI,† Fig. S22) are markedly similar to 6-thioguanosine and are assigned to the same intraligand charge transfer (ILCT), with *λ*_em_ at 390 nm and *λ*_exc_ at 326 nm. The luminescence lifetime for this ILCT was *τ* = 13 ns. At a concentration of 60 μM (Fig. 11b), the dominant feature in the spectrum of 1 remains the emission peak near 400 nm. However, the shape of the feature in the excitation–emission matrix (Fig. 11b) changes slightly, extending further to the red, which we tentatively assign to emission from aggregates.

At a higher concentration of 0.5 mM (Fig. 11c) where 1 forms vesicles, but still below the gel point, a broad unstructured emission is observed at 550 nm, which is associated with a peak in the excitation spectrum at a higher wavelength of 400 nm. This peak is assigned to a LMCT associated with a triplet state and influenced by intermolecular, aurophilic interactions within the vesicle wall (ESI,† Fig. S23). Such an interpretation is consistent with similar spectra reported for other gold complexes,^39^ including the coordination polymer [Au(6-tG)]_*n*_.^24^

Above the minimum gelation concentration c* (Fig. 11d), the broad unstructured peak at 550 nm becomes an intense peak at 490 nm which exhibits vibronic structure characteristic of an intraligand transition and indicating the loss of aurophilic interactions. Such transitions have also been reported in Au(i) complexes^40^ including the gold thiolate coordination polymer [Au(p-SPhCO_2_Me)]_*n*_.^41^ The transformation between the spectra of Fig. 11c and those of Fig. 11d is a signature of the vesicle-fibre transition studied above by microscopy. We suggest that the fibres of Scheme 1 possess a structure like the slip-stacked monomers found in the crystal structure (ESI,† Fig. S9) in which the Au(i) ions are too far apart for significant aurophilic interaction. The redshift of the emission from the gel compared to that of the free ligand can be explained in terms of phosphorescence from a triplet excited state enhanced by the metal. This interpretation is also consistent with the long lifetime (microseconds) discussed below.

**Fig. 12:**
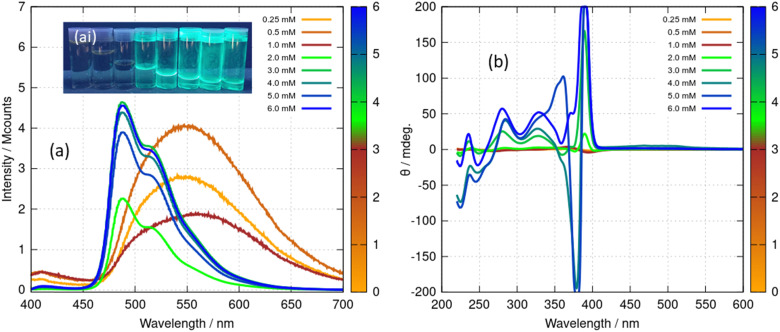
(a) Luminescence spectra of [Au(6-tGH)_2_]Cl (1) at concentrations from 0.25–6.0 mM under excitation by light of wavelength 360 nm. The data for concentrations 0.25–1.0 mM have been multiplied by 50 for clarity. (ai) The inset shows a photograph of the samples under irradiation by a mercury lamp at *λ* = 365 nm, concentration increasing left to right. (b) Circular dichroism (CD) spectra of [Au(6-tGH)_2_]Cl at concentrations from 0.25–6.0 mM. The colour bars indicate the concentration of (1) in mM.

The spectroscopic effects described above suggest that it is possible to monitor the vesicle to fibre transition near c* in more detail using photoluminescence and circular dichroism (CD). Fig. 12 shows spectra obtained for a range of concentrations above and below the transition. Those samples which had not gelled (0.25, 0.5 and 1 mM) all show the broad unstructured *λ*_max_ = 550 nm luminescence (Fig. 14a) and showed small CD signals (Fig. 12b) – consistent with Fig. 11c and with the lack of a chiral supramolecular structure to enhance the circular dichroism (CD). The CD spectrum of the monomer ([Au(6-tGH)_2_]^+^) was calculated using time-dependent density functional theory and the computed CD spectrum has similar features to the spectrum of the vesicles (ESI,† Fig. S24). This suggests that the CD signal from the vesicle is the same as the CD of the monomer apart from a bathochromic shift due to aurophilic or other intermolecular interactions. In contrast, above the gel point, there is a large increase in the ellipticity at wavelengths between 370 nm and 400 nm. This increase in CD signal occurs because the structure has changed from vesicles to fibres that take the form of a right-handed, helical ribbon (Fig. 6b). We also observed an inversion of the CD spectrum at the 370 nm and 390 nm peaks. The photoluminescence spectrum (Fig. 12a) also increases in intensity and exhibits the vibronic structure expected from Fig. 11d.

Further evidence for the effect of the vesicle-fibre transition on the optical spectra was obtained from a temperature-dependent CD experiment (ESI,† Fig. S25). Three samples of different concentrations, 2.5 mM, 3 mM, and 3.5 mM were heated. As the temperature increased, the peak near 390 nm was observed to decrease monotonically and to disappear above about 320 K as the gel breaks down. This agrees with the interpretation of the temperature-dependent ^1^H NMR data above. In confirmation of the reversible, supramolecular nature of the transition, these CD spectra showed the reformation of the gel upon cooling the sample to 293 K.

In addition to the spectral changes associated with the vesicle-fibre transition, we also observed a large change in the luminescence lifetime. ESI,† Fig. S26 shows the decay of the emission from the vesicles (at *λ* = 560 nm) and the decay of the emission from the gel (at *λ* = 490 nm). In both cases the semilogarithmic plots of the decay are non-linear, however the data is satisfactorily fitted by a model which invokes a single lifetime with a distribution owing to a variety of environments of the luminophore in these heterogeneous systems. In the case of the vesicles, the modal lifetime was 150 ns and in the case of the gel (fibres and lamellae), the modal lifetime was 57 μs. We interpret the long lifetime in the gel in terms of phosphorescence from a triplet state of the ligand influenced by the presence of the metal.

**Fig. 13:**
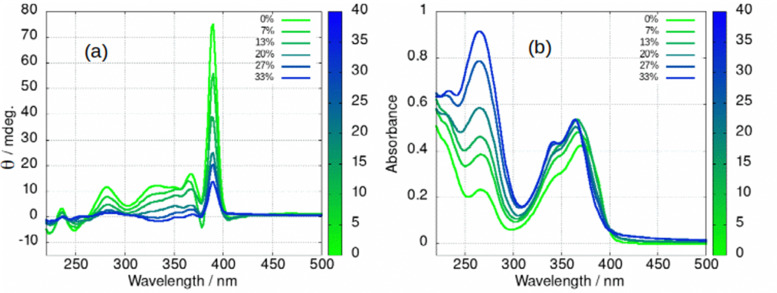
(a) Circular dichroism (CD) and (b) absorption spectrum of [Au(6-tGH)_2_]Cl(aq) at 2 mM with different percentages of acetone by volume. The colour bar indicates the volume % of acetone, 0% represents pure water as solvent.

The fibre-lamellae transition was also studied using CD spectroscopy. This transition occurs gradually at concentrations of 1 greater than about 5 mM (Scheme 1). However, it can also be induced at lower concentrations, by addition of an antisolvent which promotes unwinding of the helical ribbons as has been observed in AFM (Fig. 6). The CD spectrum of a 2 mM gel in which there are increasing percentages of acetone is shown in in Fig. 13. As the fraction of acetone is increased there is a loss of the 390 nm peak associated with the fibres (Fig. 13a). It is worth noting that the fibre to lamellae transition does not occur abruptly but shows a gradual change and the CD signal remains large even at 33% acetone. The fibres and lamellae appear to co-exist in the gel. The effect of acetone on the absorption spectrum was recorded in parallel (Fig. 13b). This shows a small hypsochromic shift of the peaks in the 350–400 nm region. As the CD signal decreases by a factor of about 7, the absorbance increases by about 30%. This provides evidence that the changes in ellipticity of Fig. 12b are due to conformational changes rather than new chemical species. It also supports the assignment of the large ellipticities to the helical ribbon structures observed in AFM (Fig. 6). A similar effect can be seen with isopropanol, (ESI,† Fig. S27), however the increasing peak at 270 nm is not present. This confirms that the increasing peak at 270 nm in Fig. 13b is simply due to the acetone absorbance and does not reflect any chemical transformation of 1.

In summary, the vesicle-fibre transition of (1) results in major changes to the photoluminescence and CD spectra: (i) the photoluminescence increases in intensity by about a factor of 50; (ii) the photoluminescence spectrum of the vesicles is broad and featureless, whereas that of the fibres is narrower and shows vibronic structure and (iii) the CD signal increases in intensity. At higher concentrations, or in the presence of antisolvents, the intense CD signal decreases as the fibres transform to lamellae. The vesicle-fibre transition occurs abruptly over a narrow range of concentrations, but in contrast the fibre-lamellae transition appears to occur gradually, and fibres and lamellae are observed together over a range of conditions.

### Cooperativity and the effect of chloride anions on the vesicle-fibre transition and gelation

The marked change in the luminescence spectra of Fig. 12a at concentrations around 1 mM is associated with the formation of a fibrous gel as shown by the analysis of the CD spectra in Fig. 12b and the microscopy of Fig. 6 and 9. This feature of 1 allows us to directly study the vesicle-fibre transition by luminescence spectra without the need for labels. First, we describe a generic equilibrium model of aggregation.

In the case of a self-assembling system at equilibrium, monomers are in equilibrium with aggregates and the aggregation of *n* monomers is described by an equilibrium constant *K*_*n*_. Such a system can be represented by a set of equilibria with values of *K*_*n*_ that depend on *n*.

1 *nM* ⇋ *M*_*n*_

**Fig. 14:**
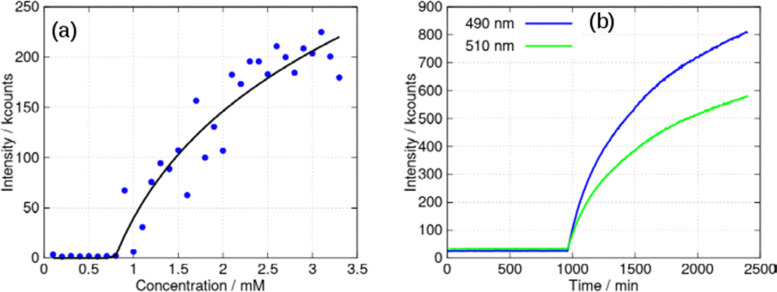
(a) Intensity of the luminescent peak at 490 nm against concentration of [Au(6-tGH)_2_]Cl (mM). The black line is the least squares fit of eqn (2) to the data. (b) Kinetic study of a 3 mM sample: luminescence intensity at two wavelengths (peaks at: 490 and 510 nm) against time. The excitation wavelength was 400 nm in all cases and spectra were acquired every 5 min for 40 hours in the kinetic study.

The aggregation may be cooperative or isodesmic. In the latter case the equilibrium constants *K*_*n*_ for the equilibria of eqn (1) are such that significant amounts of dimers, trimers and higher oligomers exist, and the fraction of the large aggregates increases gradually. In a cooperative system, *K*_*n*_ is negligible for small values of *n* and the distribution may in fact peak at a certain large value of *n*. In such cases, independent of the detailed mechanism for the cooperativity, the concentration of aggregates is negligible below a minimum concentration c* and all the additional monomers added above that concentration form aggregates. In the present case, we have two types of aggregate present (vesicles and fibres) and *K*_*n*_ may well have peaks at two different values of *n*. However, it is still clear that the fraction of monomers that are part of a fibre will be negligible below a minimum concentration. These considerations give a simple prediction for the fraction α of monomers that are part of a fibre, 
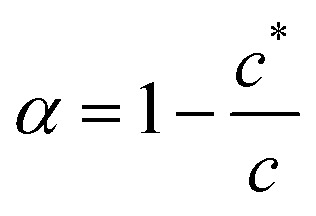
. Fig. 12a shows that the luminescence emission intensity at 490 nm is dominated by the fibres that appear upon gelation, rather than the vesicles or monomers below c*. An analysis of the concentration-dependence of the luminescence is therefore sensitive to the fraction of the sample that is present as fibres. The luminescence may also depend on the fibre concentration, but in view of inner filter effects, it is likely that the luminescence intensity is sublinear in fibre concentration. We model this part of the concentration-dependence by a factor proportional to *c*^*β*^ where 0 < *β* < 1. Fortunately, the details of this dependence are not essential to make an estimate of c* because the intensity changes abruptly at c*. Our regression model for the data of Fig. 14a is given by the fraction of monomers that are fibres multiplied by the concentration-dependence of the emission intensity of the fibres in eqn (2).2
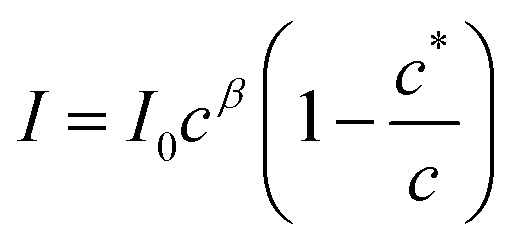


Eqn (2) is analogous to eqn (3) of a previous report,^42^ except that it is expressed in terms of concentrations rather than volume fractions. It also includes the extra factor *c*^*β*^. We estimate c* = 0.79 ± 0.1 mM for the sample of Fig. 14a. The uncertainty estimate is based on the regression analysis of the data in Fig. 14a and does not include the variation between samples. The data are consistent with an abrupt change in the concentration of fibres above c* and a cooperative model of the vesicle-fibre transition, although one should note that the kinetics of gelation are slow near c* (it takes about 18 h for a 1 mM solution to gel).

Further evidence for cooperativity comes from the data in Fig. 14b. In this experiment, a 3 mM sample of 1 was prepared and left undisturbed in a cuvette whilst luminescence spectra were recorded every 5 minutes for 40 hours. At times prior to about 960 minutes, weak luminescence corresponding to the broad, long wavelength band centred at 550 nm was observed. However, the luminescence rises suddenly after this point with simultaneous gelation of the sample. This behaviour is like well-known crystallisation phenomena, and indicative of nucleation and cooperative assembly.

**Fig. 15:**
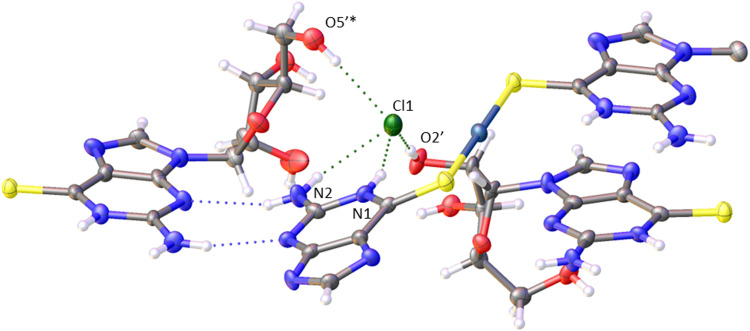
Cl^−^ hydrogen bonds in the crystal structure of [Au(6-tGH)_2_]Cl·3H_2_O.

The chloride anion plays a vital role in the formation of fibres and gelation of 1. In the crystal structure there are four major H-bonding interactions (Fig. 15). Three of these interactions are within the single chain structure as shown in Fig. 2. Two hydrogen bond donors, labelled N1 and N2, are from the same complex and two, labelled O5’* and O2′, are from sugar groups in distinct complexes. The O2′H–Cl^−^ bonds connect multiple chains.

**Fig. 16:**
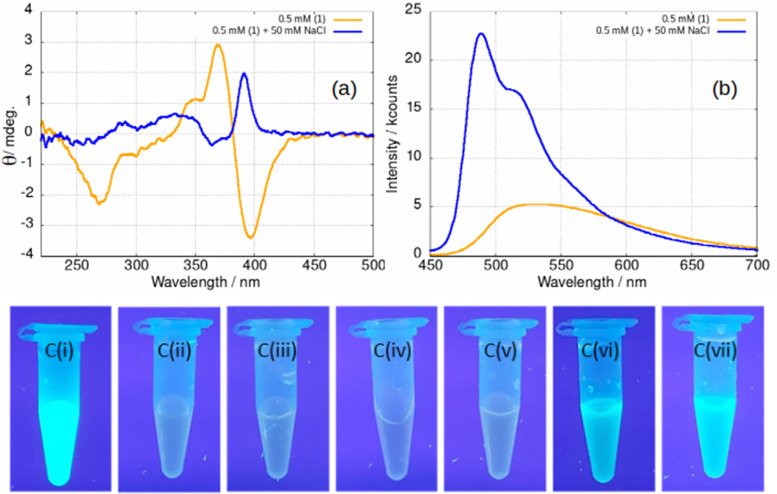
(a) CD spectrum of a 0.5 mM sample of [Au(6-tGH)_2_]Cl, 1 (orange) and a 0.5 mM sample with 50 mM NaCl added (blue). (b) Luminescence spectrum of a 0.5 mM sample (orange) and a 0.5 mM sample with 50 mM NaCl added (blue). The excitation wavelength was 410 nm. (c) Samples of 1 photographed under irradiation with a mercury lamp (*λ* = 365 nm) at various concentrations and in the presence of various aqueous salts. (i) 5 mM 1; (ii) 0.5 mM 1; (iii) 0.5 mM 1 + NaClO_4_; (iv) 0.5 mM 1 + (NH_4_)_2_SO_4_; (v) 0.5 mM 1 + NaNO_3_; (vi) 0.5 mM 1 + NaCl; (vii) 0.5 mM 1 + KCl. The salts specified in (iii)–(vii) were added to a concentration of 50 mM in every case.

Upon addition of NaCl(aq) to samples of gel, the stiffness of the gel increases and the difference between the storage and loss moduli increases (ESI,† Fig. S28). The effect is also observed in fluorescence spectra, Fig. 16. At a concentration of 0.5 mM below the minimum gelation concentration, the negative ellipticity feature at 390 nm and the broad luminescence near 550 nm are observed that have been assigned to vesicles. However, upon addition of 100 equivalents of NaCl(aq) a positive ellipticity is observed at 390 nm and the luminescence spectra changes to that characteristic of the fibres (Fig. 16a and b).

The effect of NaCl(aq) is associated with Cl^−^ and not ionic strength or the cation as can be seen from Fig. 16c. A series of samples of gel under irradiation are shown with the leftmost a positive control of 1 at a concentration of 5 mM above the gel point and the next a negative control at 0.5 mM below the gel point. Sample Ci is brightly luminescent and sample Cii is weakly luminescent as expected. Successive photographs Ciii–Cvii show samples of 1 at a concentration of 0.5 mM in the presence of 50 mM of a selection of aqueous salts. Only in the case of samples Cvi and Cvii, which contain NaCl(aq) and KCl(aq), is the characteristic luminescence of the fibrous gel (Fig. 12a) observed. In some cases, the gel is not as homogenous as observed in Fig. 16c vi–vii and forms droplets of luminescent material suspended in the solution. This tends to happen at low chloride ion concentrations. We suggest that this indicates a situation in which the concentration of fibres is insufficient to form a sample-spanning hydrogel (ESI,† Fig. S29).

## Conclusion

A gold(i) coordination complex of 6-thioguanosine, [Au(6-tGH)_2_]^+^, has been prepared and its structure determined by single crystal X-ray diffraction. The metal nucleoside complex has a linear coordination geometry and is an example of a bolaamphile with polar headgroups (sugar moieties) on either side of the hydrophobic thionucleobase-Au(i) core. The sugars are responsible for the chirality of the complex and the metal-thionucleobase is responsible for its luminescence. In aqueous media [Au(6-tGH)_2_]Cl, demonstrates a rich self-assembly phenomenology. Vesicles are observed below about 1 mM in which aurophilic interactions between neighbouring complexes in the vesicle wall led to a broad, structureless luminescence at about 550 nm with a lifetime of 150 ns. At the minimum gelation concentration near 1 mM, a sharp transition occurs to fibres and the system forms a metallo-hydrogel. AFM images and CD spectroscopy provide evidence that the fibres have the form of helical ribbons. At the gel point, the luminescence spectrum gains the features of a ligand centred phosphorescence (vibronic structure and a lifetime of 57 μs). At higher concentration (>5 mM) or upon the addition of antisolvents (acetone or isopropanol) the helical ribbons transform to lamellae. This occurs with a marked decrease in the intensity of the CD spectrum as the helical ribbons are replaced by lamellae. If the antisolvent is introduced in a gradual manner by vapour diffusion, the lamellae can assemble to form crystals.

The luminescence and chiral properties of 1 allowed the observation of the vesicle-fibre and fibre-lamellae transitions by spectroscopic means. The vesicle-fibre transition occurs simultaneously with gelation and AFM images near the gel point show the presence of vesicles connected by strands of single molecular dimension. This suggests a mechanism for the vesicle-fibre transition in which collisions between vesicles lead to two vesicles becoming connected by a thin fibre. We hypothesize that this mechanism is more general for such vesicle-fibre transformations. The abrupt concentration-dependence and the time-dependence of the transition are more consistent with a cooperative rather than an isodesmic model of the fibre formation process. The crystal structure shows chloride acting as a hydrogen bond acceptor and halide ions were also found to be important for the gelation - the minimum gelation concentration is lowered in the presence of excess aqueous chloride. In contrast, the fibre-lamellae transition does not appear to occur at a sharply defined concentration, but lamellae of monomolecular thickness can be prepared for examination by probe microscopy.

## Experimental

### Preparation of [Au(6-tGH)_2_]Cl at 10 mM concentration

The reaction to make [Au(6-tGH)_2_]Cl proceeds in two steps. In the first step HAuCl_4_ (chloroauric acid; 9.5 mg; 2.80 × 10^−2^ mmol) is reduced from Au(iii) to Au(i) by thiodiglycol (5.59 μL; 5.59 × 10^−2^ mmol) in water (1 mL) as described in our previous work.^24^ In the second step 6-thioguanosine (16.7 mg; 5.59 × 10^−2^ mmol) is suspended in 1.79 mL of water *via* sonication and then vigorous stirring. The coordination takes place through the addition of the Au(i) solution from step one to the stirred suspension of 6-thioguanosine. The solution turned from a dark, cloudy yellow to a pale clear yellow over 10 minutes while stirring continued. Signs of gelation became apparent within 1 h. ^1^H NMR (500 MHz, deuterium oxide, 363 K) *δ* 8.89 (s, 2H), 6.49 (d, *J* = 5.1 Hz, 2H), 5.25 (t, *J* = 5.2 Hz, 2H), 4.95 (t, *J* = 5.0 Hz, 2H), 4.78 (td, *J* = 4.7, 3.4 Hz, 2H), 4.41 (ddd, *J* = 12.6, 4.7, 3.5 Hz, 4H). ^1^H NMR (500 MHz, DMSO-d_6_, 298 K) *δ* 12.65 (s, 1H), 8.38 (s, 1H), 7.36 (s, 2H), 5.74 (d, *J* = 5.5 Hz, 1H), 4.41 (t, *J* = 5.3 Hz, 1H), 4.12 (*dd*, *J* = 4.9, 3.8 Hz, 1H), 3.91 (*q*, *J* = 3.9 Hz, 1H), 3.60 (ddd, *J* = 46.0, 12.0, 4.0 Hz, 2H). IR selected data (cm^−1^): 1652 (m) (NH_2_ bend), 1580 (l), 1562(l). ES-MS: *m*/*z* (positive mode) found 795.1042 (calcd. for [Au(6-tGH)_2_]^+^ 795.1).

### Preparation of samples at other concentrations proceeded in analogous fashion

#### Crystallisation of [Au(6-tGH)_2_]Cl·3H_2_O

A hydrogel of [Au(6-tGH)_2_]Cl (10 mM) was used for vapour diffusion, with acetone as an antisolvent. After about two weeks, colourless needle crystals had formed in the gel, which were suitable for single-crystal X-ray diffraction (XRD). After a month, the vapour diffusion transformed all the gel into crystals. The crystals were collected and tested using powder X-ray diffraction. Crystals were also grown using isopropanol as an antisolvent, and these crystalline solids showed the same PXRD pattern as the acetone-grown crystals. The melting point of the crystals was found to be 356 K.

## Materials and methods

Reagents were obtained from Sigma-Aldrich and used as-received. NANOpure® deionized water (nominal 18.2 MΩ cm resistivity) was obtained from a NANOpure® DIamond™ Life Science ultrapure water system equipped with a DIamond™ RO Reverse Osmosis System (Barnstead International).

### Spectroscopy

UV-Vis absorption spectra were collected on a NanoDrop™ One/OneC Microvolume UV-Vis Spectrophotometer using 3 μL of a sample at a variety of concentrations.

Fluorescence microscopy was performed on a Shimadzu (RF-6000) UV/Vis Spectrofluorophotometer using a 500 μL quartz cuvette.

Luminescence spectra and excited-state lifetimes were measured using an Edinburgh FLS980 photoluminescence spectrometer, equipped with a 450 W Xenon arc lamp, Czerny Turner excitation and emission monochromators (1.8 nm mm^−1^ dispersion; 1800 grooves per mm), time-correlated single photon counting (TCSPC) module and a Hamamatsu R928 P photomultiplier tube (in a fan assisted TE cooled housing, operating temperature −20 °C). Time-resolved measurements up to 10 μs were performed using the TCSPC option; a picosecond pulsed diode laser (EPL-375; 370.8 nm; 61.1 ps pulse width) with repetition rates between 10 kHz and 1 MHz was used to excite the samples. For excited-state lifetimes >10 μs a different experimental setup was used, by equipping the spectrometer with a phosphorescence module and a 60 W xenon flash lamp (μF920H, full-width at half maximum, FWHM = 1 μs) with a variable flash rate between 0.1 Hz and 100 Hz); the signals were collected using a multi-channel scaling (MCS) card in the photon-counting module.

^1^H and ^13^C NMR spectra were collected on a Bruker 500 Avance III HD NMR spectrometer operating at 500.15 MHz and 125.78 MHz, respectively. All shifts are quoted in ppm relative to tetramethylsilane. The solvents used were deuterium oxide (D_2_O) and deuterated dimethyl sulfoxide (DMSO-d_6_).

FTIR spectra (in the range 500–4000 cm^−1^) were recorded in transmission mode on a Shimadzu IR Affinity-1S with an ATR attachment. 50 scans were co-added and averaged, and the resolution was 1 cm^−1^.

Electrospray mass spectrometry was collected on a Waters Xevo G2-XS Quadrapole Time-of-Flight (QToF) Mass analyser. The sample was directly infused using the fluidics system. Sample concentration approx. 5–10 μM. Capillary – 3.0 kV; Cone – 40 V; Offset – 80 V; Source Block Temp – 110 °C; Desolvation Gas Temp – 300 °C; Cone Gas Flow – 50 L h^−1^; Desolvation Gas Flow – 600 L h^−1^; MS Scan Speed – 0.5 s Polarity – Positive Ion Mode; Scan Mode – Full Scan (100–1200 Da); Ion Source – Electrospray Ionistation.

Circular dichroism (CD) spectra were recorded on a Jasco J-810 spectrometer under temperature control. All samples were recorded in a demountable quartz cuvette (Hellma) of path length 0.1 mm.

### Rheology

Rheological measurements were performed with a HR-2 Discovery Hybrid Rheometer (TA Instruments) with a standard steel parallel-plate geometry of 20 mm diameter with a gap of 1 mm. The strain and the frequency were set to 1% and 1 Hz during the frequency and time sweeps respectively.

### Microscopy

#### Electron microscopy (SEM/TEM)

Scanning electron microscopy of samples was performed using a Tescan Vega LMU scanning electron microscope (Tescan, Cambridge, UK) housed within EM Research Services, Newcastle University. Digital images were collected with Tescan-supplied software. The samples were dehydrated by freeze-drying and mounted on an aluminium stub using carbon tape. The samples were then coated with gold, 5–10 nm, using a Polaron SEM Coating Unit (Quorum Technologies Ltd, Lewes, UK) prior to imaging.

Transmission electron microscopy was performed after uranyl acetate staining of the samples. 10 μL of solution was settled for 1 minute on a pioloform-coated (Agar Scientific Ltd, UK) copper mesh grid (Gilder Grids, Grantham, UK) and dried under a lamp. The grids were examined on a Hitachi HT7800 transmission electron microscope using an Emsis Xarosa camera with Radius software by EM Research Services, Newcastle University.

### Atomic force microscopy (AFM)

A Multimode-8 atomic force microscope with Nanoscope V controller and ‘‘E’’ scanners (Bruker) was used for acquiring AFM data. An isolation table (AMETEK TMC) was used to reduce vibrational noise. Nanoscope software version 9.1 was used to control the microscope. The system was operated in a peak force tapping mode at ultra-low forces (order of pN) to minimise deformation or damage to the samples. The AFM data were processed with NanoScope Analysis 1.50 software (Bruker).

For AFM in air, silicon tips on silicon nitride cantilevers (ScanAsyst, Bruker) were used for collecting images. The nominal tip radius was approximately 2 nm. The cantilever backside was coated with aluminium, resonant frequency 70 kHz, and spring constant *k* about 0.40 N m1. The system was operated in ScanAsyst in air mode (peak force tapping mode). All samples for AFM in air were deposited on silicon chips.

For *in situ* AFM (under liquid), the experiments were performed in an AFM fluid cell (MTFML-V2, Bruker). Silicon tips on silicon nitride cantilevers (SNL, Bruker) were used for imaging. The nominal tip radius was approximately 2 nm. The cantilever backside was coated with Ti/Au, resonant frequency 50–80 kHz, and spring constant *k* about 0.35 N m1. The system was operated in ScanAsyst in fluid mode (peak force tapping mode). The fluid cell was mounted on a precleaned silicon chip. The fluid cell chamber was filled with an aqueous solution of the sample.

The silicon chips were sonicated in acetone and left to dry overnight. Once dry, they were cleaned in piranha solution (a mixture of 30% H_2_O_2_ and 98% sulfuric in the ratio 3 : 7 by volume). [Caution: piranha solution is a strong oxidizing agent and has been known to detonate spontaneously upon contact with organic material; it should be handled with extreme caution]. After removal of the piranha solution, they were washed with deionized water and sonicated in deionized water, then sonicated in acetone. The chips were stored in acetone until use when one is removed from the acetone with tweezers and dried under nitrogen.

### X-Ray diffraction (XRD)

#### Powder diffraction

Powder XRD was used to determine the bulk material by matching the experimental diffraction pattern to the predicted diffraction pattern based on the single crystal structure data. The diffractometer used was a XRD3 – Bruker D2 Phaser with LynxEye detector. The sample was transferred directly on to the zero-background holder without grinding or drop casting. A preliminary scan between 5–120° 2theta was run to check for low angle peaks prior to main measurement. Diffractometer setup: 5–80° 2theta, 0.05 step, 5 s per step, divergence slit = 0.6 mm.

#### Single crystal diffraction

A single crystal suitable for XRD was grown using vapour diffusion of an antisolvent (acetone) into a 10 mM aqueous solution of the sample. Crystallisation took approximately two weeks and produced clear needles, MP 356 K. Crystal Data for C_20_H_26_AuClN_10_O_8_S_2_ (*M* =885.09 g mol^−1^): monoclinic, space group *P*2_1_ (no. 4), *a* = 7.3414(3) Å, *b* = 13.5389(5) Å, *c* = 14.8400(6) Å, *β* = 101.962(4)°, *V* = 1442.98(10) Å^3^, *Z* = 2, *T* = 150.0(2) K, *μ*(CuKα) = 12.470 mm^−1^, *D*_calc_ = 2.037 g cm^−3^, 20519 reflections measured (8.932° ≤ 2*θ* ≤ 134.216°), 5112 unique (*R*_int_ = 0.0857, *R*_sigma_ = 0.0717) which were used in all calculations. The final *R*_1_ was 0.0391 (*I* > 2*σ*(*I*)) and w*R*_2_ was 0.0927 (all data).

The scattering is dominated by the gold atom in this structure and hence the hydrogen atoms could not be located using peaks in the Fourier difference map. They were added where expected based on the hybridisation of the parent atoms and the balance of charges in the structure. Where appropriate the DFIX card was applied to ensure the hydrogen bond network was consistent.

Crystal structure data for [Au(6-tGH)_2_]Cl was collected on a Xcalibur, Atlas, Gemini ultra diffractometer equipped with a fine-focus sealed X-ray tube (*λ* CuKα = 1.54184 Å) and an Oxford Cryosystems CryostreamPlus open-flow N_2_ cooling device. Cell refinement, data collection and data reduction were undertaken *via* software CrysAlisPro (Rigaku OD, 2015). Intensities were corrected for absorption empirically using spherical harmonics. The structure was solved using XT^43^ and refined by XL^44^ through the Olex2 interface.^45^

## Data availability

The data that support the findings of this study are openly available at the following URL/DOI: https://doi.org/10.25405/data.ncl.23899113.

## Author contributions

Synthetic work, PXRD, spectroscopic and rheological characterisation was carried out by LFMcG. Microscopy was carried out by OEZ and LFMcG. Single crystal X-ray diffraction was performed by PW and the PL investigations by FC. The work was supervised by AH and BRH. The manuscript was written by BRH, AH and LFMcG. All coauthors have edited and/or approved the manuscript.

## Conflicts of interest

There are no conflicts to declare.

## Supplementary Material

SM-019-D3SM01006F-s001

SM-019-D3SM01006F-s002
