# Supplementary material for: Vesicles, fibres, films and crystals: A low-molecular-weight-gelator [Au(6-thioguanosine)2]Cl which exhibits a co-operative anion-induced transition from vesicles to a fibrous metallo-hydrogel
Source: Soft Matter. 2023 Oct 19;19(43):8386–402. doi: 10.1039/d3sm01006f (PMC10630954; doi:10.1039/d3sm01006f)
Supplement: SM-019-D3SM01006F-s001 [file SM-019-D3SM01006F-s001.pdf]

Electronic Supplementary Material (ESI) for Soft Matter.
This journal is © The Royal Society of Chemistry 2023

# Vesicles, Fibres, Films and Crystals: A Low-Molecular-Weight-Gelator [Au(6-thioguanosine)$_2$]Cl Which Exhibits a Co-operative Anion Induced Transition from Vesicles to a Fibrous Metallo-Hydrogel.

*Liam F. M^c^Garry, Osama El-Zubir, Paul G. Waddell, Fabio Cucinotta, Benjamin R. Horrocks and Andrew Houlton.*

Chemical Nanoscience Laboratory, Chemistry, School of Natural and Environmental Sciences,

Newcastle University, Newcastle upon Tyne, UK, NE1 7RU

**Supporting Information**

**Contents**

**1. [Au(6-tGH)$_2$]Cl (1) characterisation data**

Figure S1 – high resolution mass spectrometry.

Figure S2 – comparison of PXRD of a polycrystalline sample of [Au(6-tGH)$_2$]Cl with the powder pattern computed from the single crystal XRD structure.

**2. Vesicles**

Figure S3 – additional AFM images of fibres forming between vesicles.

**3. Lamellae and crystals**

Figure S4 – electron micrographs of the xerogel formed from 6 mM gels.

Figure S5 – electron micrographs of the xerogel after addition of acetone.

Figure S6 – Powder X-ray diffraction data for the xerogel.

Figure S7 – Powder X-ray diffraction data for crystals made by addition of isopropanol.

Figure S8 – Schematic of the relevant crystal planes.

Figure S9 – Olex2 images of the crystal structure of [Au(6-tGH)$_2$]Cl.3$H_2O$.

Figure S10 – Schematic of the vapour diffusion method for crystallization from the xerogel.

Figure S11 – AFM images of crystals forming after vapour in-diffusion of acetone.

Figure S12 – Optical micrographs of crystallization.

**4. Gelation**

Figure S13 – Inversion test and heating / cooling of the gel.

Figure S14 – Rheology of the gel.

Figure S15 – Temperature-dependent $^{1}H$ NMR spectra of the gel.

**5. NMR spectroscopy**

Figure S16 – $^{1}H$ NMR spectrum at 363 K with proton assignments to the molecule.

Figure S17 – $^{1}H$ NMR COSY spectrum at 363 K – sugar proton correlations.

Figure S18 – $^{1}H$–$^{13}C$ HSQC data at 363 K.

Figure S19 – $^{1}H$ NMR spectra at 298 K with peaks due to 2,2'-sulfinyldiethanol indicated.

Figure S20 – $^{1}H$ NMR COSY spectrum at 363 K with correlations due to protons on 2,2'-sulfinyldiethanol indicated.

**6. Optical spectroscopy**

Figure S21 – UV-Vis absorption spectra at high dilution.

Figure S22 – Photoluminescence spectra at high dilution.

Figure S23 – Schematic model of the vesicles.

Figure S24 – Calculated and experimental CD spectra.

Figure S25 – Temperature-dependent CD spectra.

Figure S26 – Luminescence decay curves below and above the minimum gelation concentration.

Figure S27 – CD spectra after addition of isopropanol antisolvent.

**7. Evidence of the chloride ion effect on gelation**

Figure S28 – Rheological data for the chloride anion effect.

Figure S29 – Optical micrographs showing the chloride anion effect.

**8. Au-S bond lengths for coordinated thione and thiolate ligands**

Literature structural data on C-S bond lengths

**1. [Au(6-tGH)$_2$]Cl characterisation data**

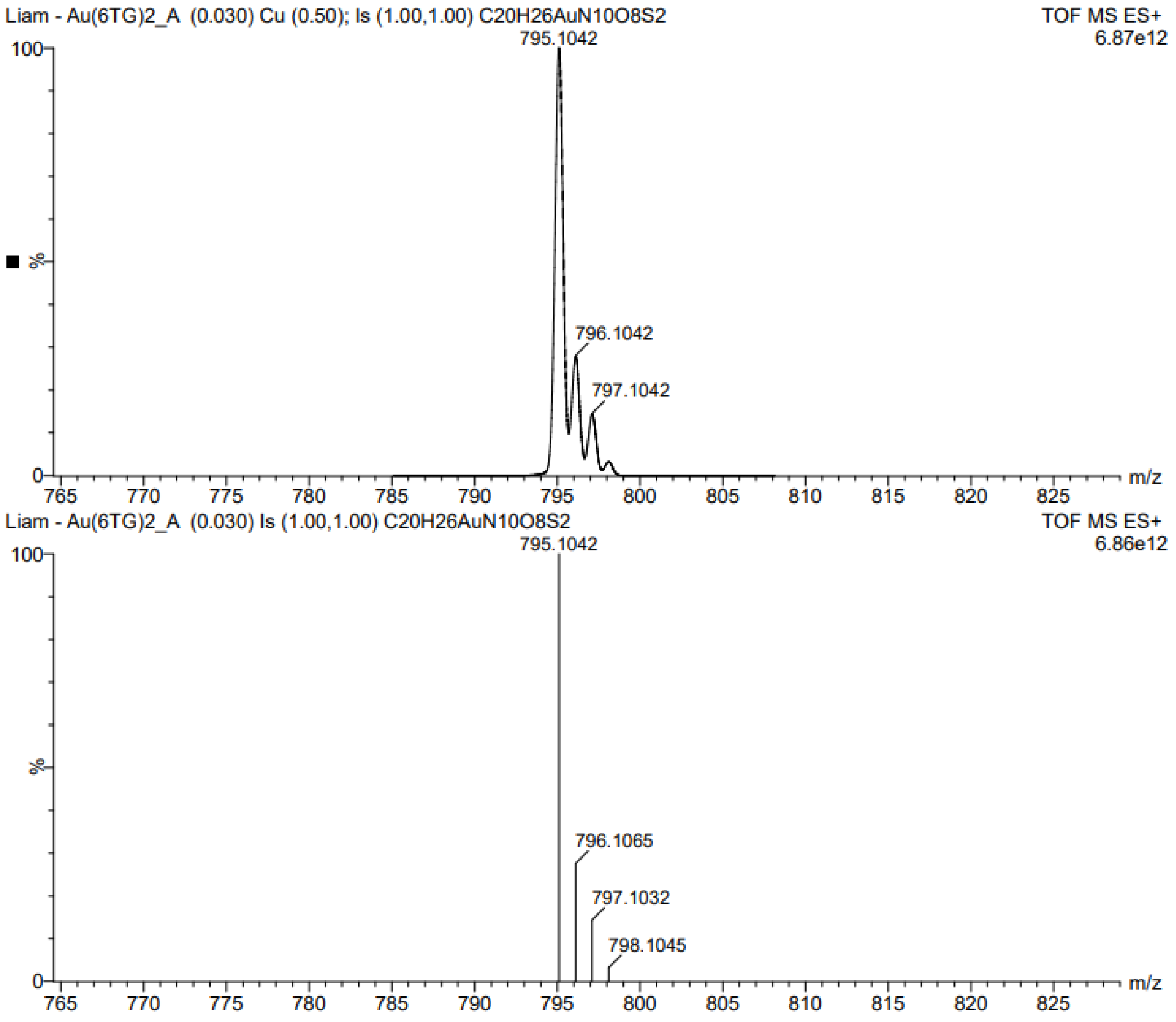


**Figure S1**: Electrospray mass spectrometry (ESI-MS) in positive ion mode of [Au(6-tGH)$_2$]$^+$(aq) (top) experimental data and (bottom) predicted spectrum.

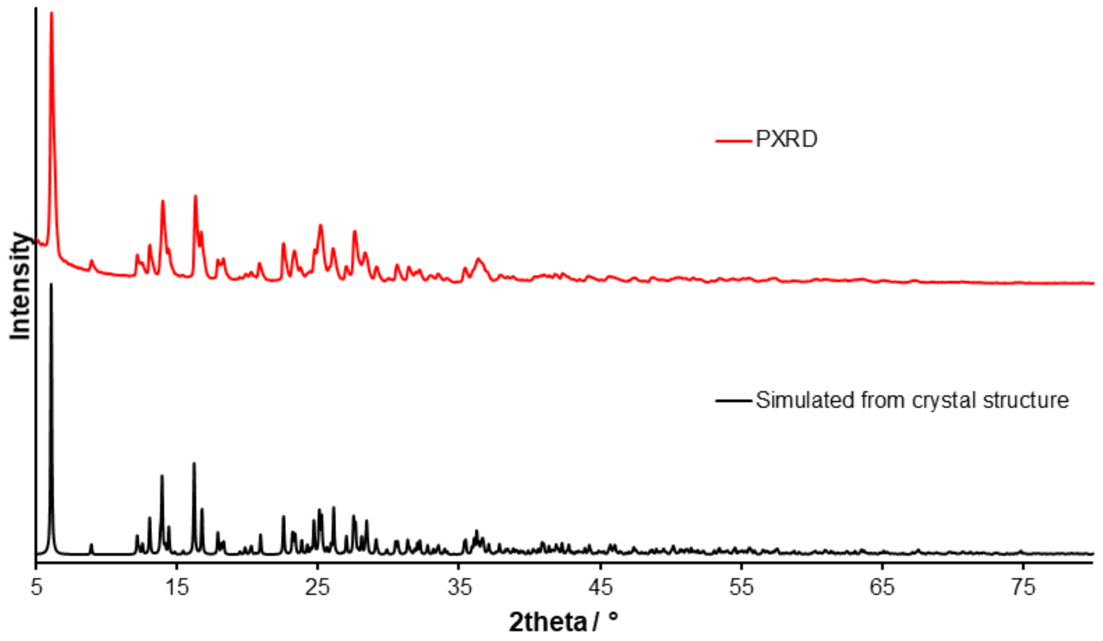


**Figure S2**: Experimental PXRD of $[Au(6\text{-}tGH)_2]Cl$ (red line) compared to the PXRD pattern calculated from the crystal structure (black line).

**2. Vesicles**

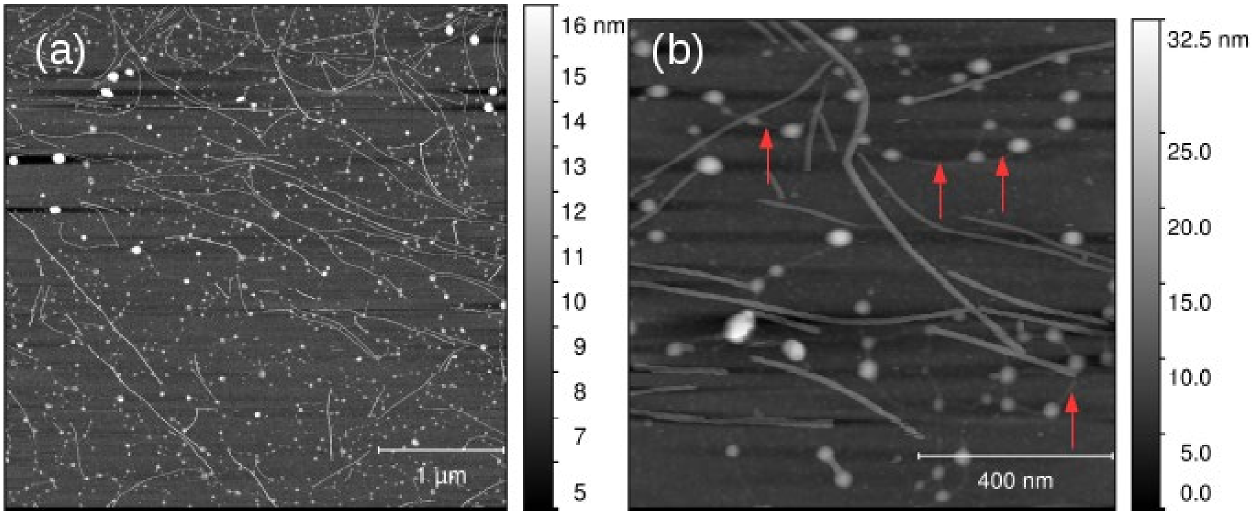


**Figure S3**: Two in situ AFM images at 1 mM of $[Au(6\text{-}tGH)_2]Cl$ showing the vesicles (bright spots), fibres and the molecular strands forming between vesicles (examples indicated by red arrows in image (b)).

**3. Lamellae and crystals**

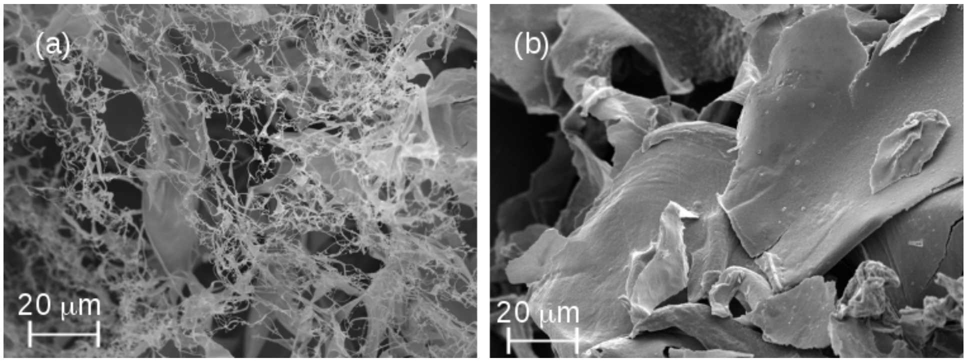


**Figure S4**: Scanning electron micrograph (SEM) of a xerogel obtained from a freeze-dried gel of [Au(6-tGH)$_2$]Cl (**1**). Formed at (a) 2 mM and (b) 6 mM. At the lower concentration (2 mM) fibres are observed. At higher concentrations (6 mM) mainly sheets rather than fibres are observed.

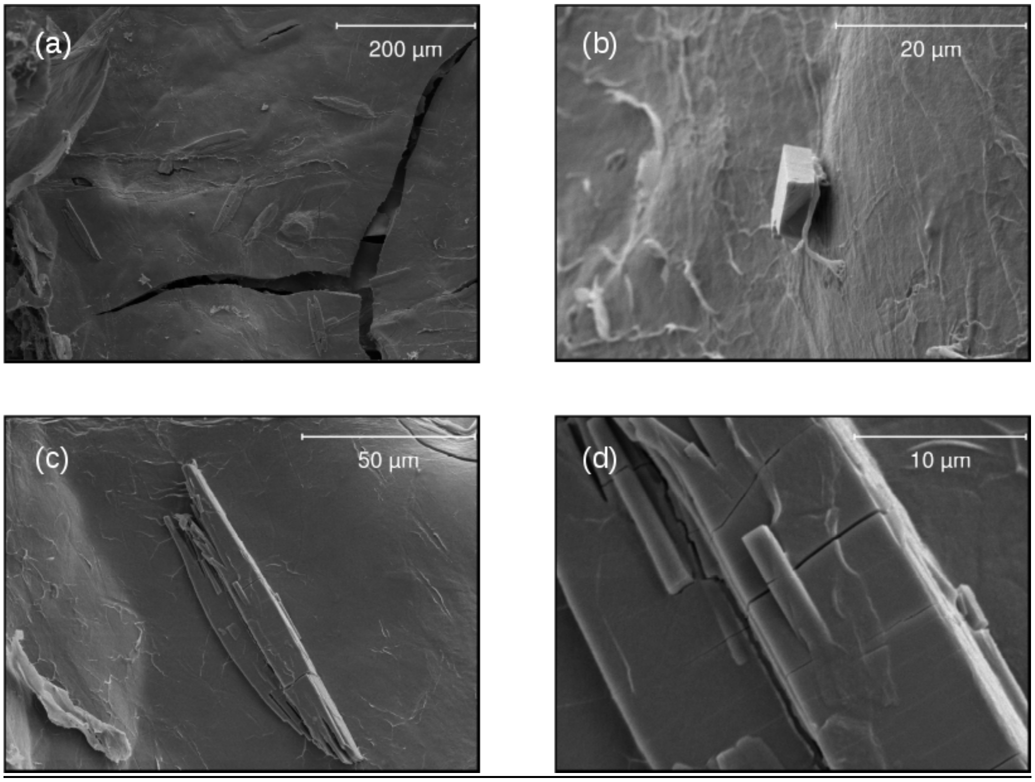


**Figure S5**: SEM images of films of [Au(6-tGH)$_2$]Cl made by addition of an equal volume of acetone to a 10 mM gel. Lamellae and crystals are observed.

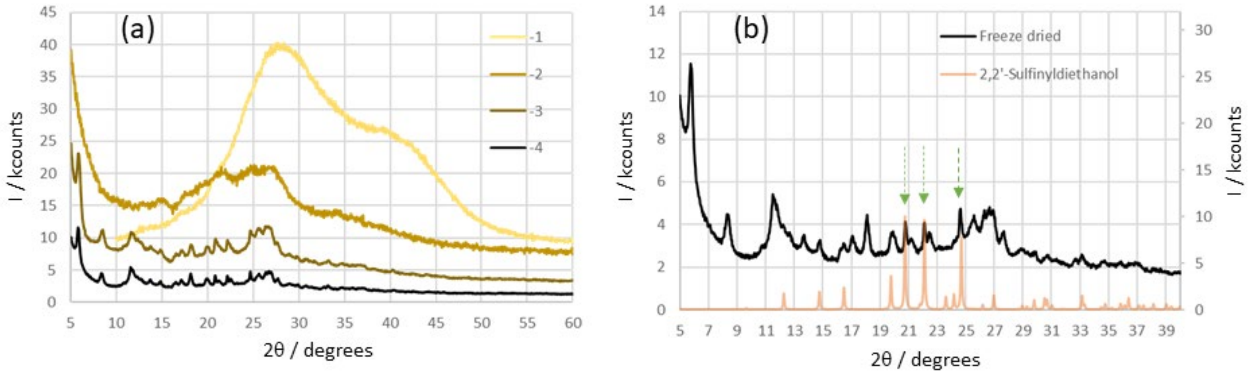


**Figure S6**: (a) PXRD of *c* = 10 mM $[Au(6\text{-}tGH)_2]Cl$ xerogels. (1) Xerogel before freeze drying; (2) after freeze drying; (3) further drying under vacuum for one week and (4) after drying under vacuum for two weeks. (b) The xerogel made by freeze drying and two weeks of vacuum drying (black line) compared to PXRD for the oxidation product of the reducing agent used to prepare Au(I); 2,2-sulfinyldiethanol. The three most intense peaks from the 2,2-sulfinyldiethanol pattern are indicated by green arrows.

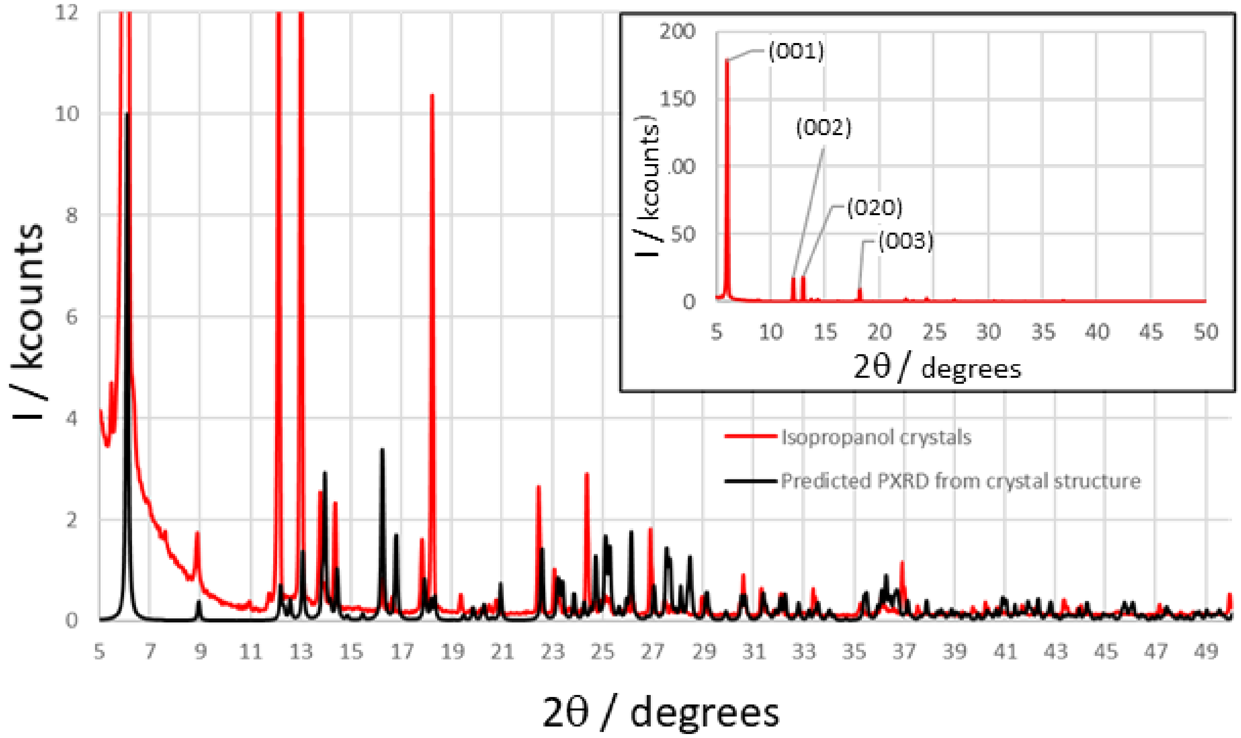


**Figure S7**: PXRD of isopropanol grown crystals (red line) and the predicted PXRD from the crystal structure (black line). Inset: the isopropanol crystal PXRD on a larger scale with the peaks corresponding to (001); (002); (020) and (003) reflections labeled.

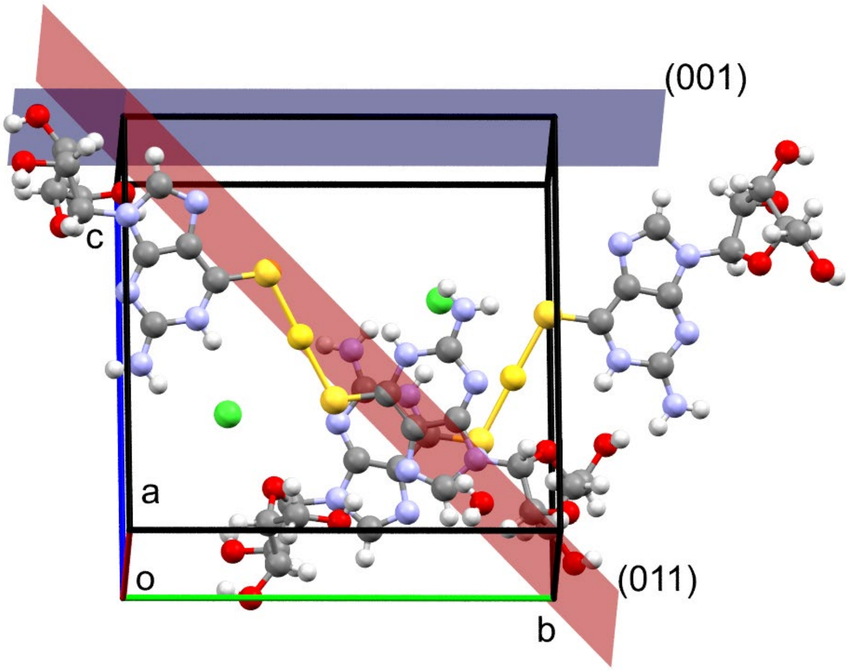


**Figure S8**: The crystal planes (001) and (011).

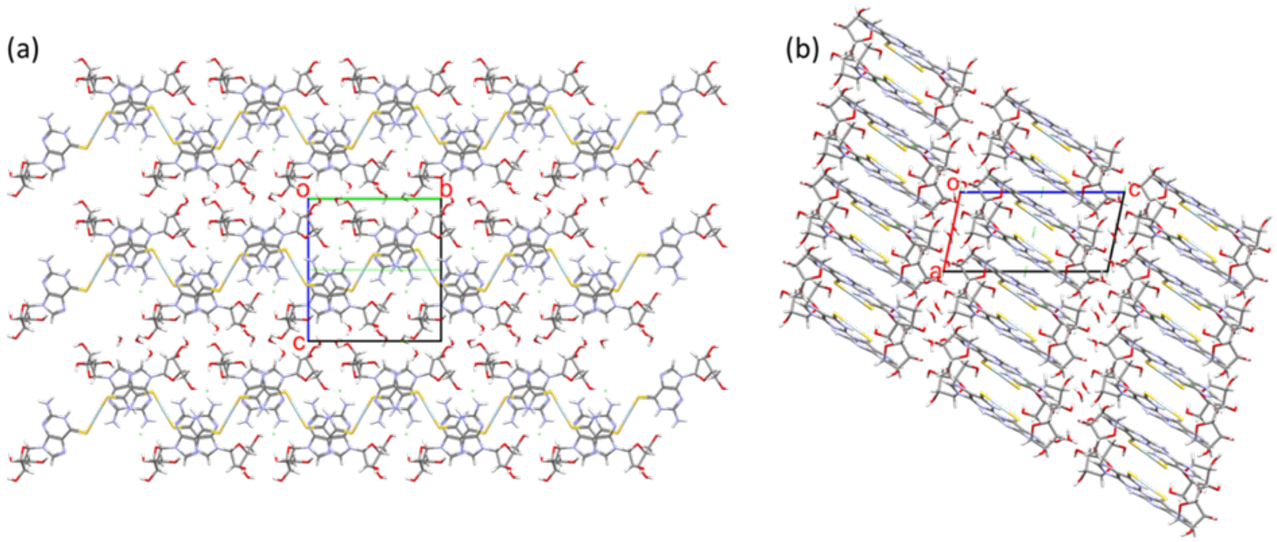


**Figure S9**: Olex2 images of the crystal structure of **1**. (a) a view normal to the *cb* plane of the unit cell that shows the chain alignment in the crystal. (b) A view normal to the *ac* plane of the unit cell showing the chain stacking.

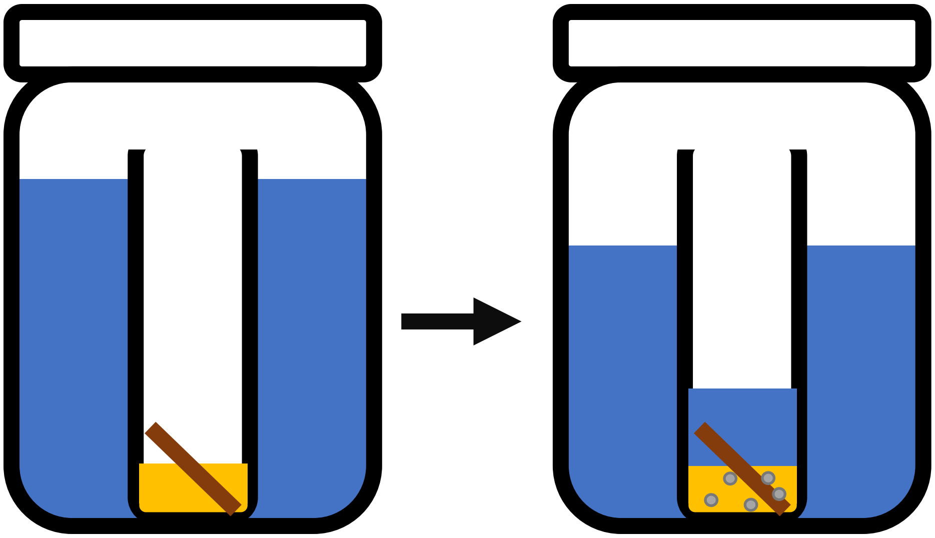

**Figure S10**: Vapor diffusion setup for crystallization of **1** on silicon chips. The blue antisolvent (acetone or isopropanol) diffuses slowly in to the inner vial where the gel (yellow) and the silicon (brown line) are. After a few days (acetone) or a few weeks (isopropanol) crystals start to form (grey dots) in the gel. The silicon can then be removed and analyzed using AFM to observe the crystal growth. The remaining crystals can be left to grow and then analyzed using XRD.

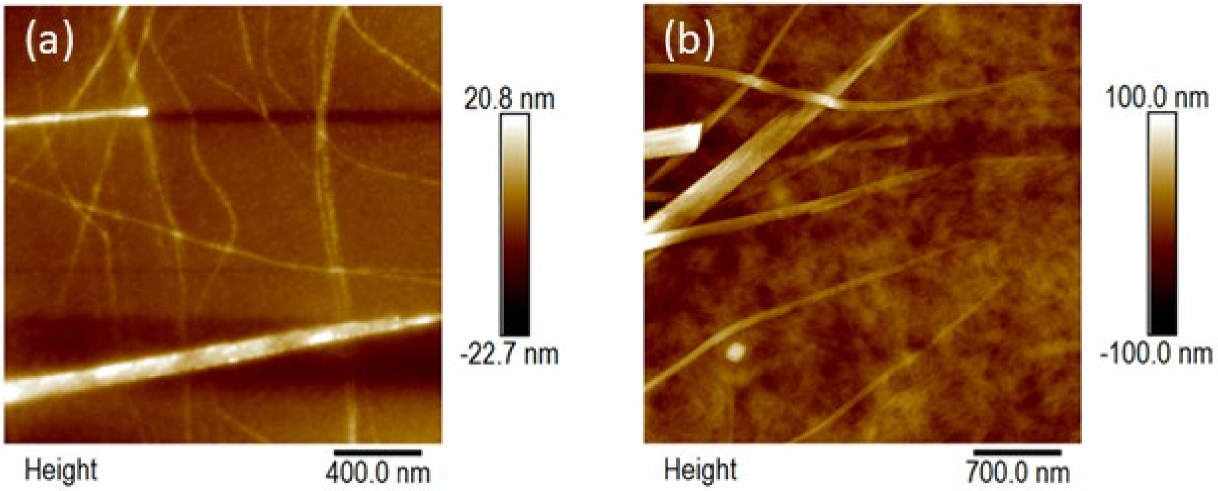


**Figure S11**: AFM images of $c$ = 5 mM $[Au(6\text{-}tGH)_2]Cl$ on silicon. Acetone was vapour-diffused into the sample as the antisolvent. (a) individual fibres and rigid, needle-shaped crystals. (b) crystals (top left) forming on top of the gel, visible in the background as a dense network of fibres.

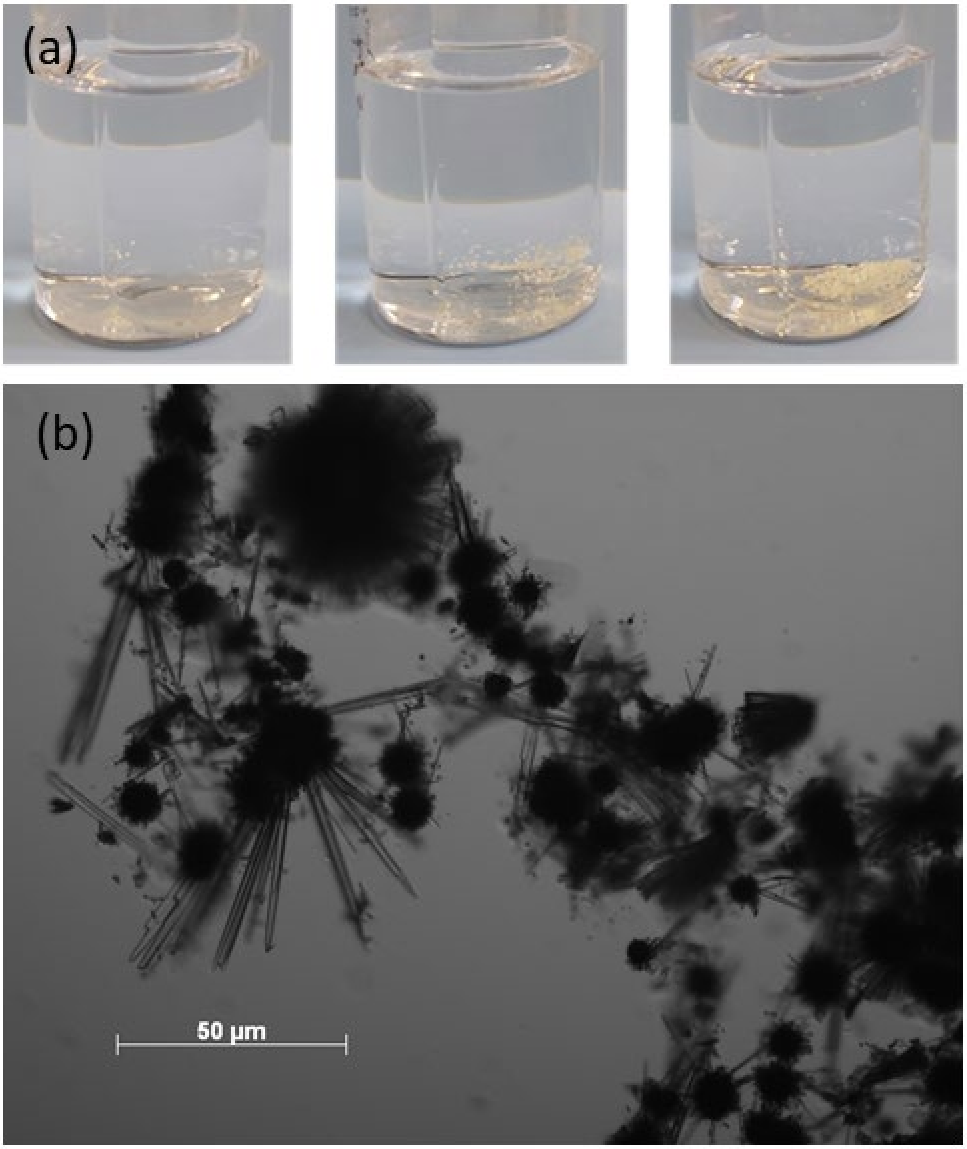


**Figure S12:** (a) Photographs showing the transformation from gel to crystals (left to right) during vapor diffusion of acetone into a 10 mM gel. (b) Optical micrograph of the crystals of **1** at 40x magnification.

**4. Gelation**

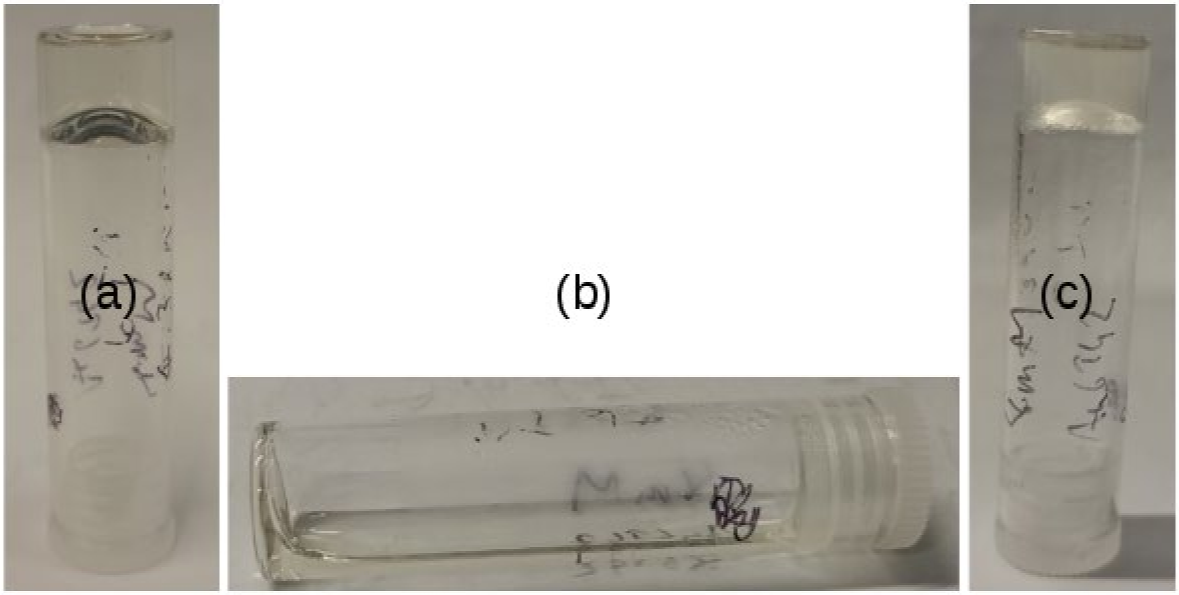


**Figure S13**: Inversion test on a 4 mM aqueous sample of $[Au(6\text{-}tGH)_2]Cl$: (a) before, (b) during, and (c) after heating.

(a) Sample at room temperature in the gel state.

(b) The sample heated to 100$^{o}$C and in the liquid state.

(c) The sample after cooling for 48 h to room temperature. Gel has reformed.

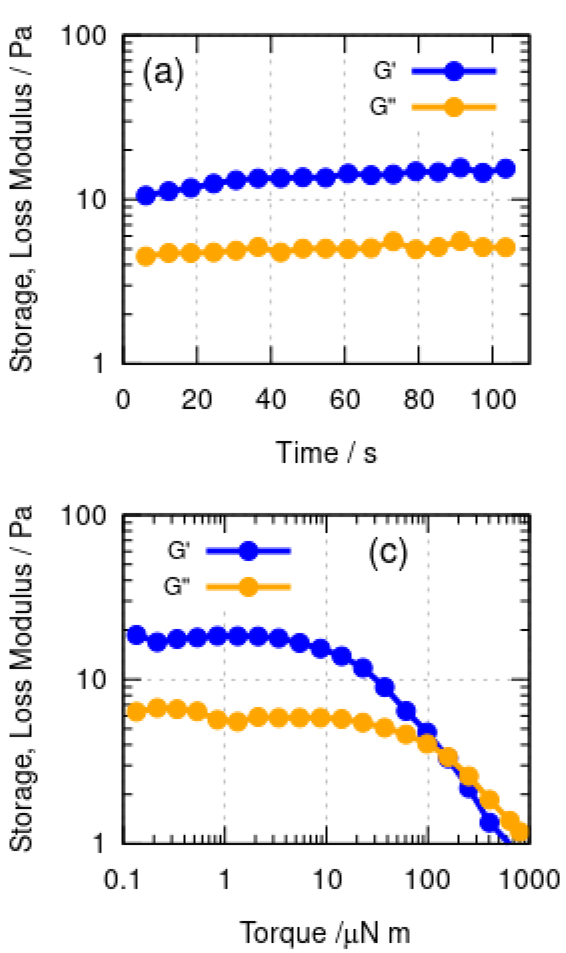


**Figure S14**: Rheological study of a 10 mM sample 24 h after preparation. (a) Time sweep (frequency = 1 Hz); (b) frequency sweep; (c) amplitude (torque) sweep and (d) viscosity against shear rate.

## 5. NMR spectroscopy

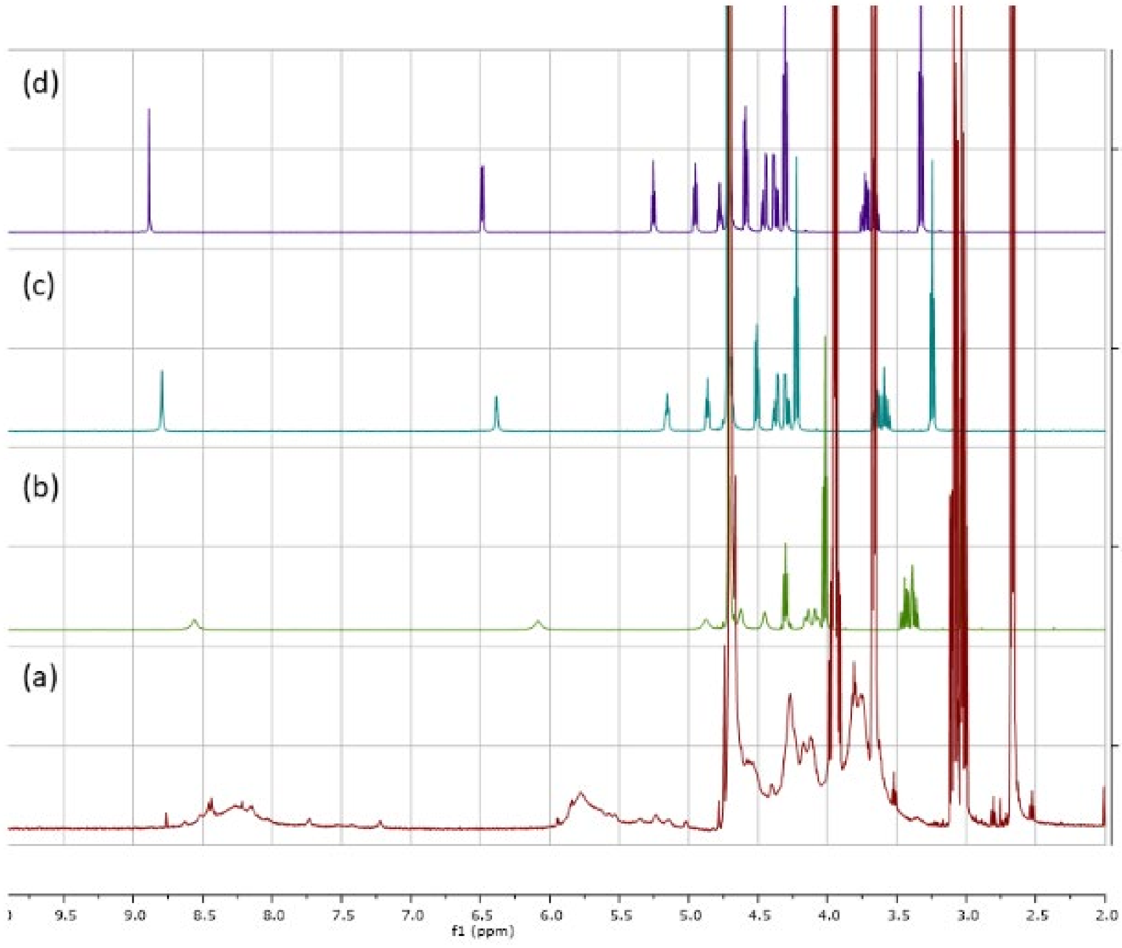


**Figure S15**: $^{1}$H NMR of a 4.5 mM sample of [Au(6-tGH)$_2$]Cl in $D_2O$ recorded on a 500 MHz instrument at (a) 298 K, (b) 333 K, (c) 353 K, and (d) 363 K.

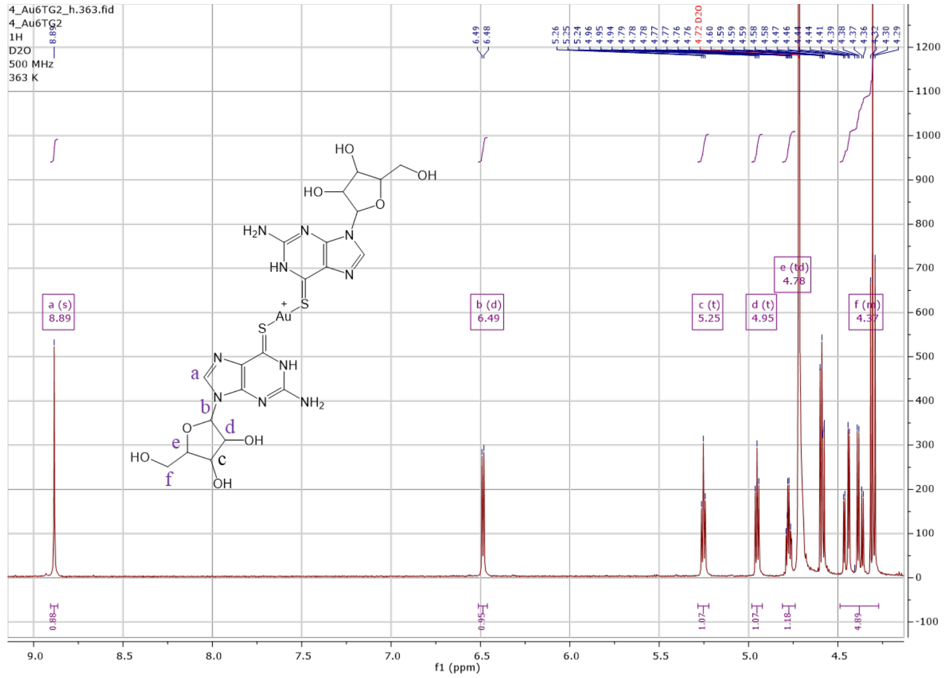


**Figure S16**: $^{1}$H NMR of 4.5 mM sample of $[Au(6\text{-}tGH)_2]Cl$ in $D_2O$ recorded on a 500 MHz at 363 K with proton assignments to molecule.

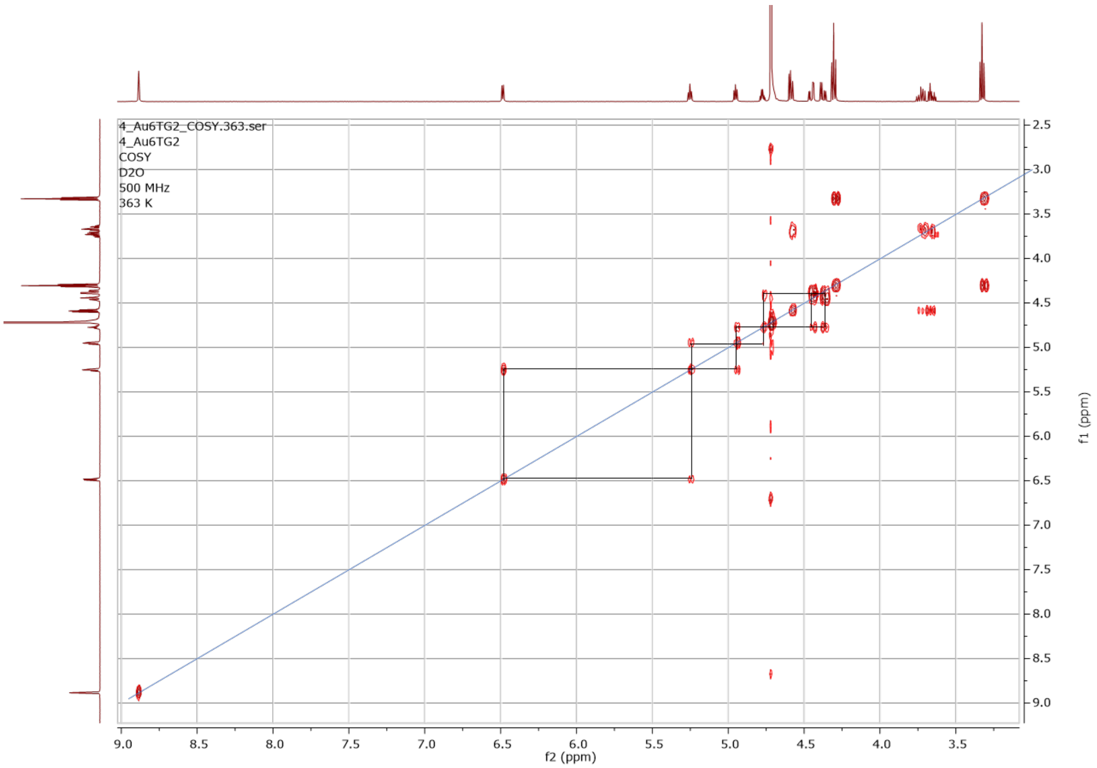


**Figure S17**: COSY spectrum of 4.5 mM sample of $[Au(6\text{-}tGH)_2]Cl$ in $D_2O$ recorded on a 500 MHz instrument at 363 K with the correlations of sugar protons indicated.

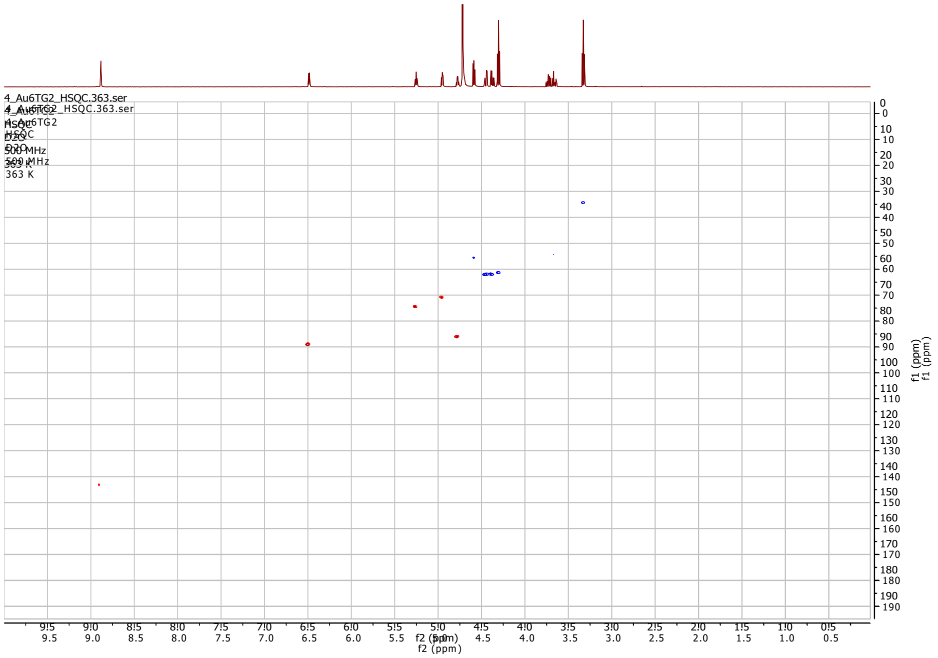


**Figure S18**: $^{1}H$–$^{13}C$ HSQC data for 4.5 mM sample of $[Au(6\text{-}tGH)_2]Cl$ in $D_2O$ recorded on a 500 MHz instrument at 363 K.

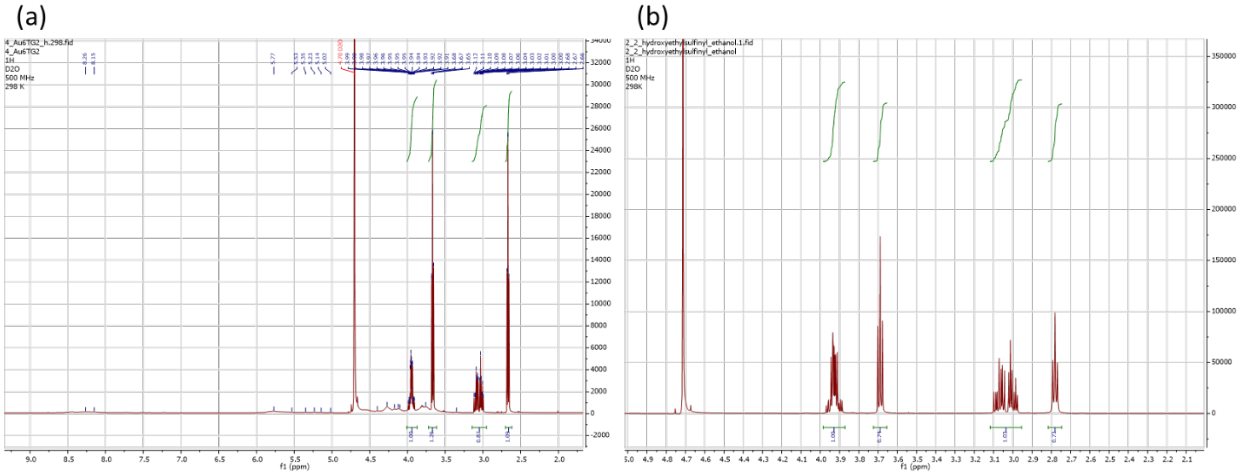


**Figure S19**: (a) The four peaks from the oxidation product of the reducing agent (2,2'-sulfinyldiethanol), used to prepare Au(I), show prominently in the $^{1}H$ NMR of a 4.5 mM sample in $D_2O$ recorded on a 500 MHz at 298 K. (b) $^{1}H$ NMR of the reactant mixture from the first step of the coordination reaction in $D_2O$ recorded on a 500 MHz instrument: chloroauric acid ($HAuCl_4$) and 2,2'-thiodiglycol. These form Au(I) and 2,2'-sulfinyldiethanol which can sometimes be crystallised under alternative crystallisation conditions. The four peaks from the oxidation product of the reducing agent, 2,2'-sulfinyldiethanol remain sharp at 298 K confirming that it is not part of the supramolecular structure of $[Au(6\text{-}tGH)_2]Cl$, **1**.

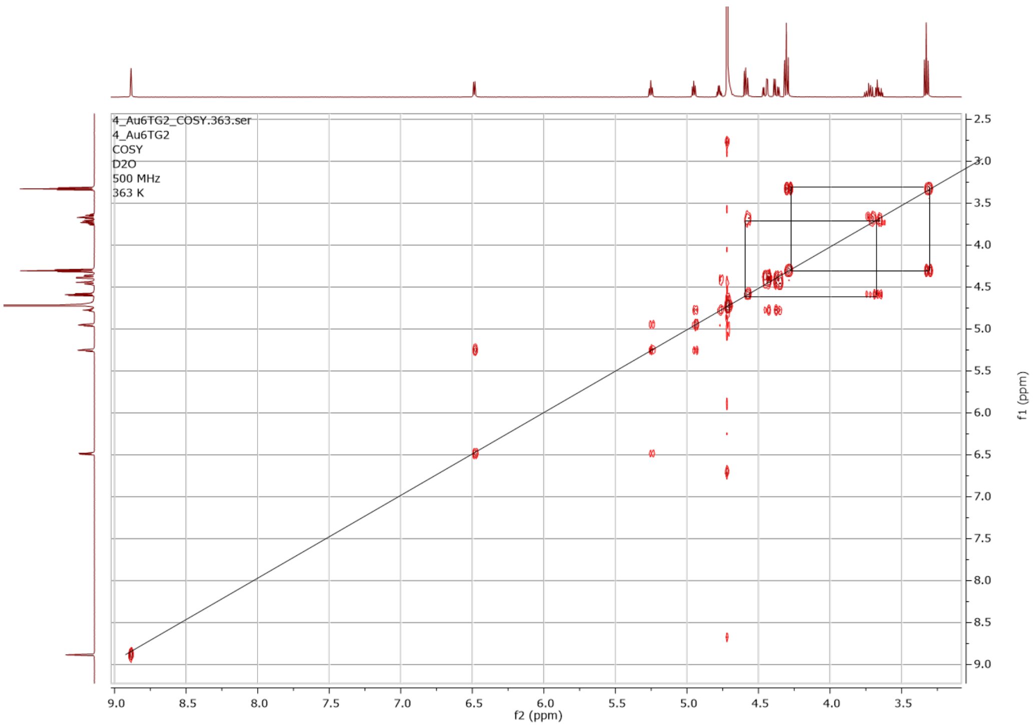


**Figure S20**: COSY spectrum of 4.5 mM sample of [Au(6-tGH)$_2$]Cl in $D_2O$ recorded on a 500 MHz at 363 K with correlations of protons from 2,2'-sulfinyldiethanol indicated.

6. **Optical spectroscopy**

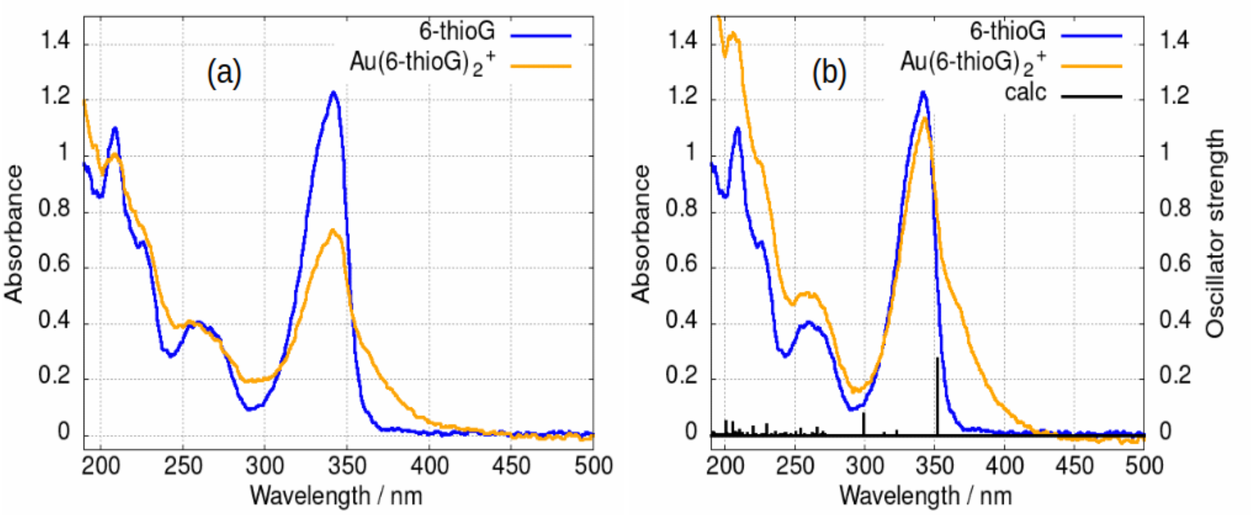


**Figure S21**: UV-Vis absorption spectra. (a) 6-thioG 60 μM (blue line) and after addition of Au(I) (orange line). The ligand:metal stoichiometry is 2:1. (b) both ligand 6-thioG (blue line) and $[Au(6\text{-}tGH)_2]Cl$ (orange line) at a concentration of 60 μM. 6-thioG $\lambda_{max}$ = 344 nm, ε = 2.0 x $10^4$ $M^{-1}cm^{-1}$. $[Au(6\text{-}tGH)_2]Cl$ $\lambda_{max}$ = 344 nm, ε = 1.8 x $10^4$ $M^{-1}cm^{-1}$. The results of a TD-DFT calculation of the vertical excitation energies and oscillator strengths for $[Au(6\text{-}tGH)_2]^+$ are shown in black. The calculation used the B3LYP functional with the 6-31G(d,p) basis set for the non-metal atoms and the LanL2DZ basis set for Au.

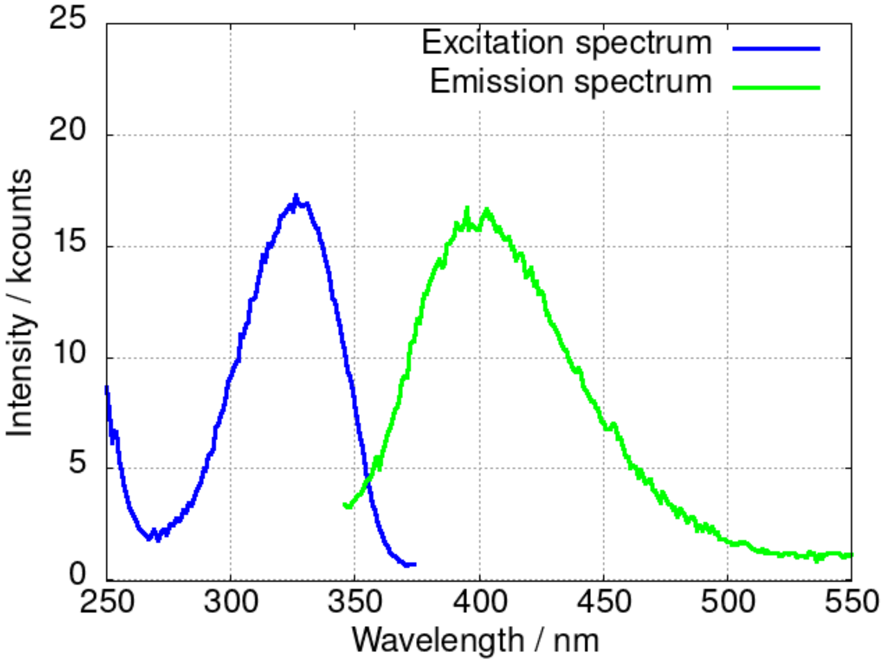


**Figure S22**: Excitation and emission spectra of **1** at 5 μM concentration. The excitation wavelength was $\lambda_{exc}$ = 300 nm for the emission spectrum and the emission wavelength $\lambda_{em}$ = 440 nm for the excitation spectrum.

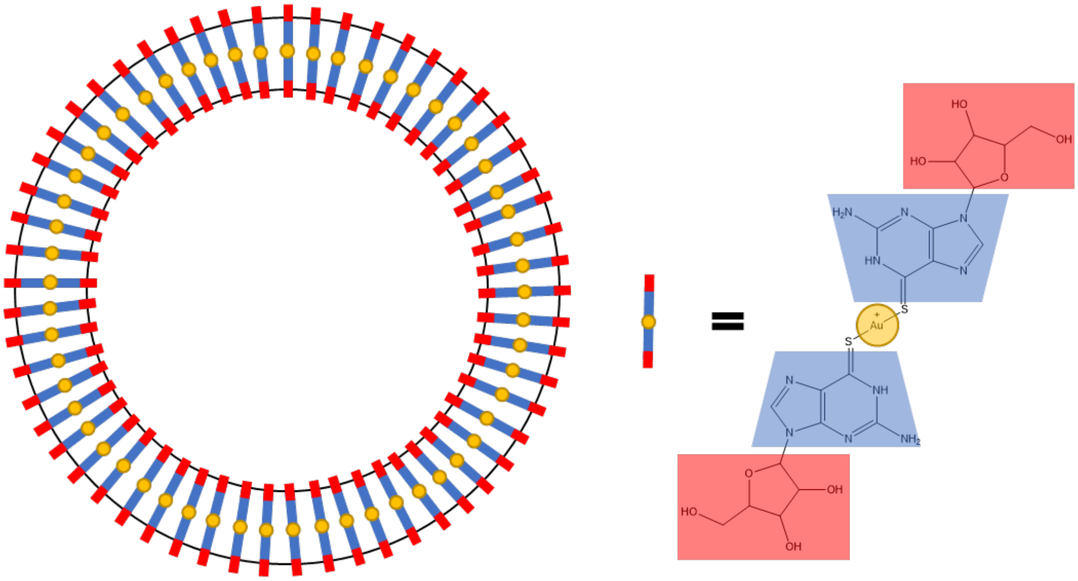


**Figure S23**: Model of **1** monomers in the vesicle wall. The red areas indicate the hydrophilic sugar groups which form the wetted internal and external surfaces. The blue areas indicate the hydrophobic nucleobases and yellow indicates the Au ions. The alignment of the Au ions facilitates aurophilic interactions.

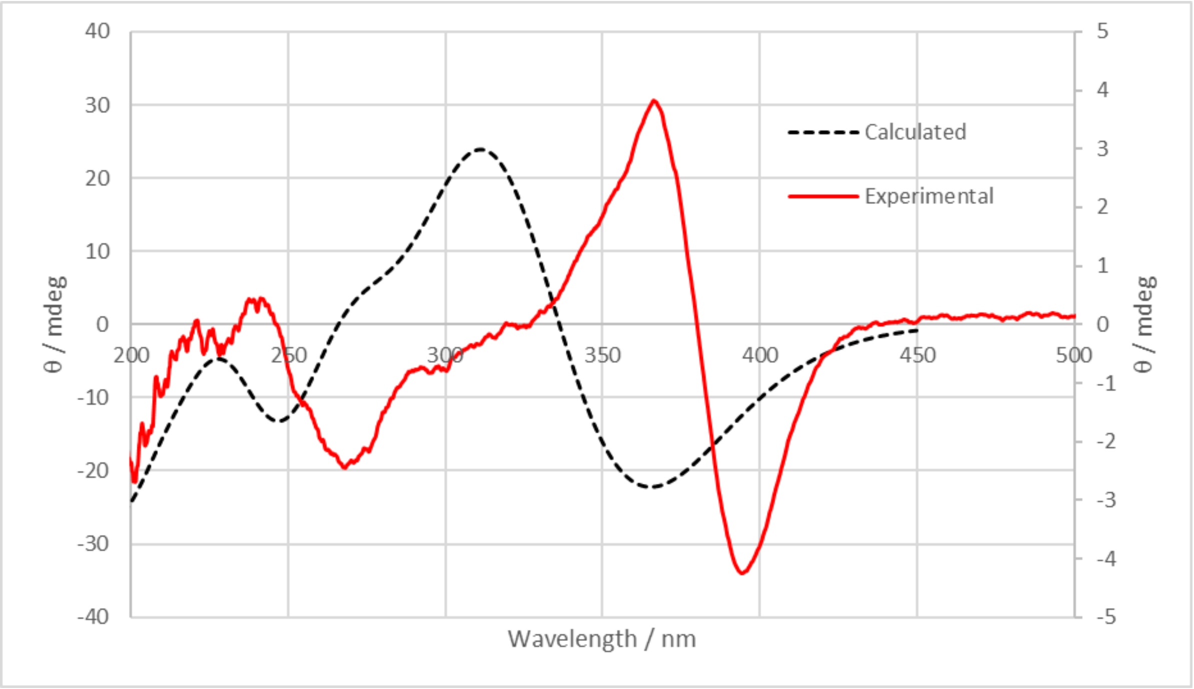


**Figure S24**: Calculated CD spectrum of monomeric $[Au(6\text{-}tGH)_2]^+$ using the B3LYP functional and the 6-31G(d,p) basis set for the non-metallic atoms and the LanL2DZ basis set for Au (dashed black line). Experimental CD data for $[Au(6\text{-}tGH)_2]Cl$ just under the gel point at 1 mM (red line).

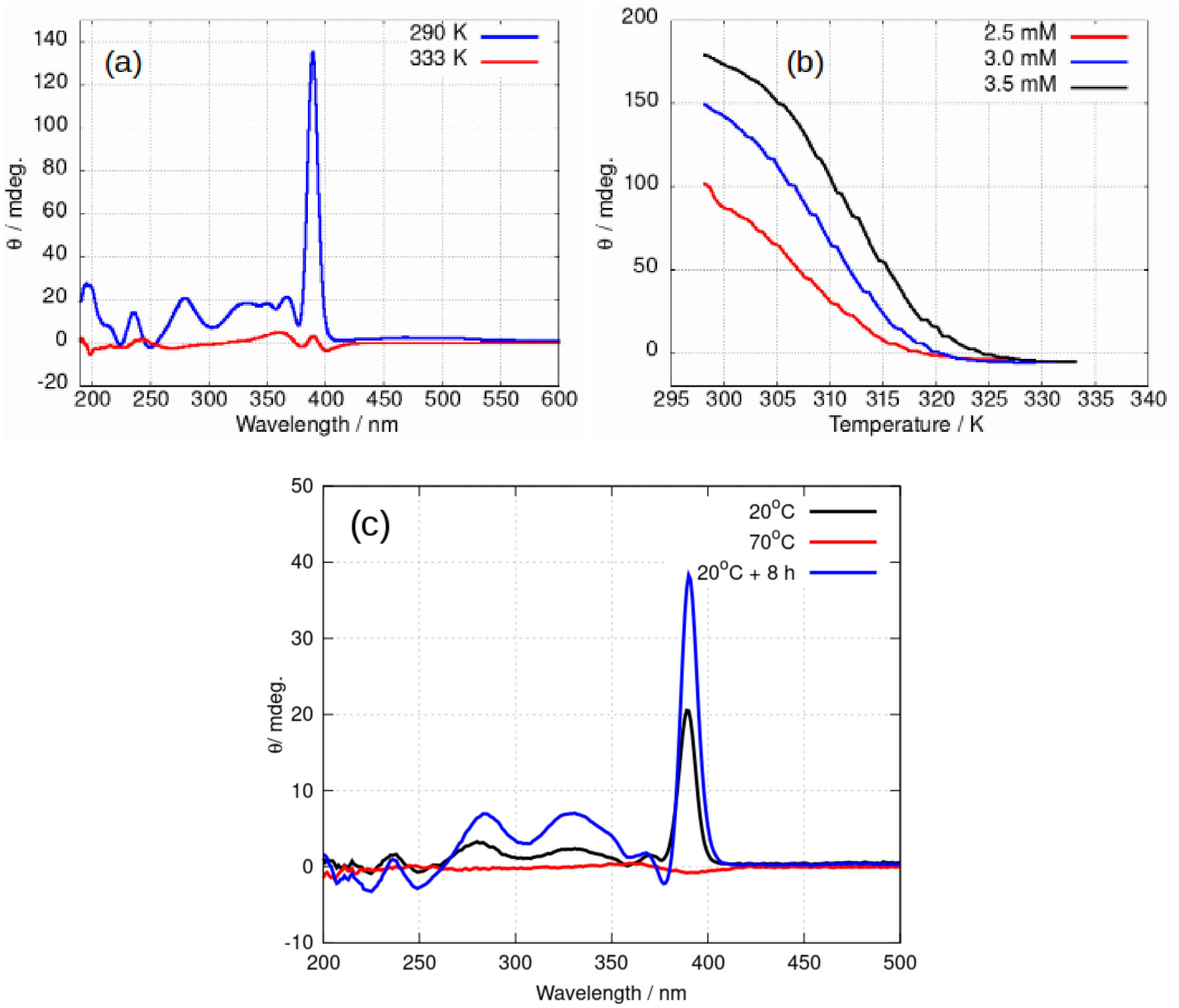


**Figure S25**: CD spectra at different temperatures. (a) 3 mM of [Au(6-tGH)$_2$]Cl at room temperature, about 290K (blue line) and at 333 K (grey line). (b) ellipticity at 390 nm for samples at 2.5, 3.0 and 3.5 mM concentrations of aqueous [Au(6-tGH)$_2$]Cl. (c) Heat-cool cycle for a 4 mM gel sample. The black line is the spectrum of the gel at 20°C before heating, the red line shows the spectrum recorded at 70°C and the blue line shows the spectrum after cooling to 20°C for 8 h.

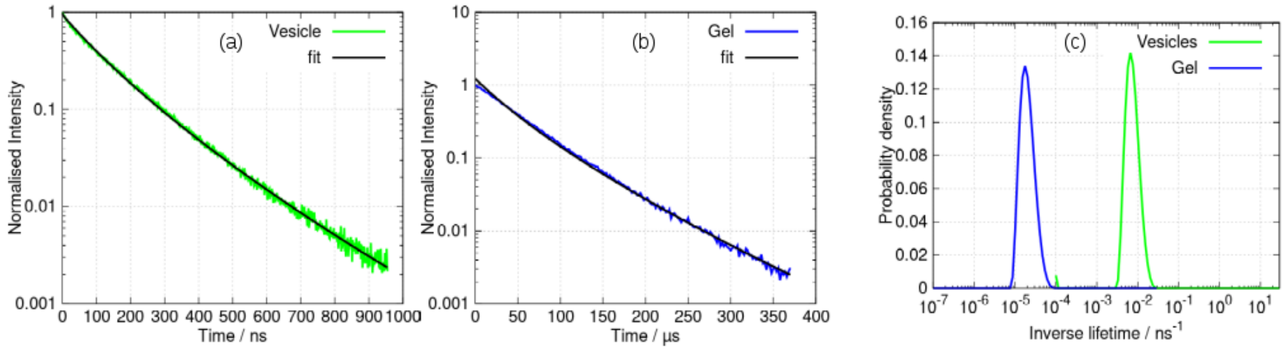


**Figure S26**: Luminescence decay curves below and above the minimum gelation concentration (mgc). (a) Vesicles of 0.5 mM (**1**) below the mgc ($\lambda_{exc}$ = 375 nm, $\lambda_{em}$ = 560 nm); (b) the gel at 4 mM ($\lambda_{exc}$ = 375 nm, $\lambda_{em}$ = 490 nm) and (c) the distribution of lifetimes extracted from the fits to the decay curves.

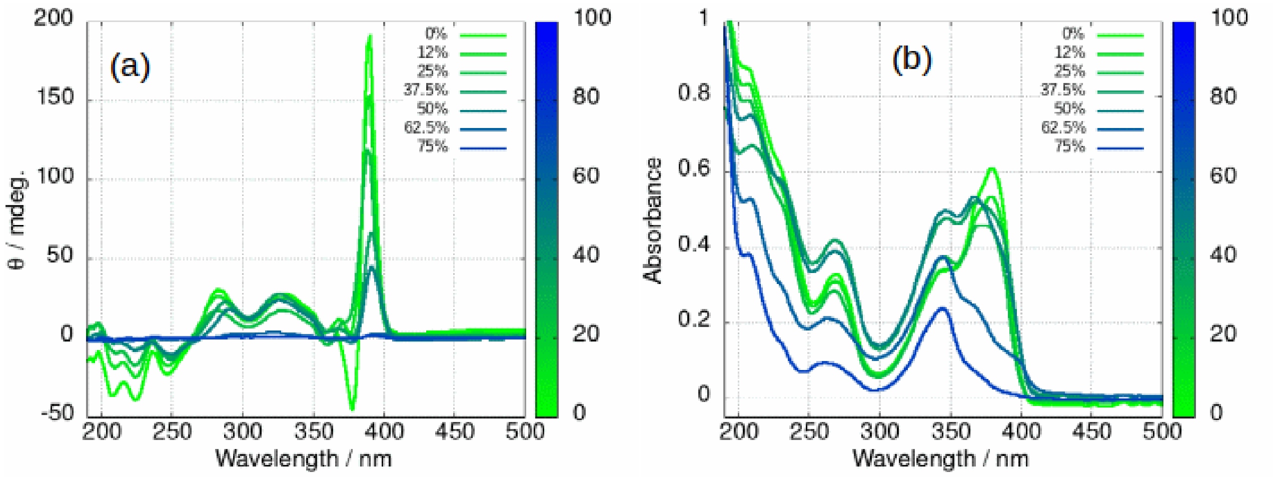


**Figure S27**: (a) Circular dichroism spectra and (b) absorbance spectra of $[Au(6\text{-}tGH)_2]Cl$ gels at a concentration of 3 mM in isopropanol/water mixtures. The colour scale indicates the volume percentage of isopropanol.

**7. Evidence of the chloride ion effect on gelation**

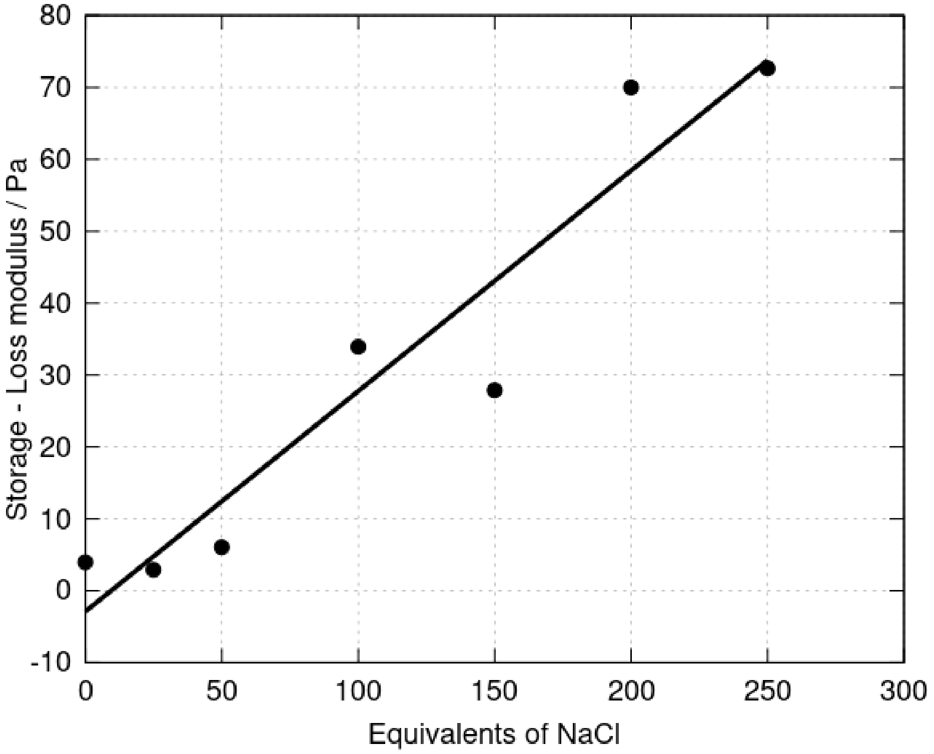


**Figure S28:** Difference between the storage (G') and loss (G') moduli for gels prepared from a 4 mM sample of $[Au(6\text{-}tGH)_2]Cl$ with the addition of excess NaCl. The concentration of NaCl is reported as equivalents in terms of Au. The storage and loss moduli were measured at 1 Hz frequency and the mean values over a period of 176 s are shown. A linear correlation of this measure of stiffness with equivalents of chloride observed ($r^2 = 0.91$).

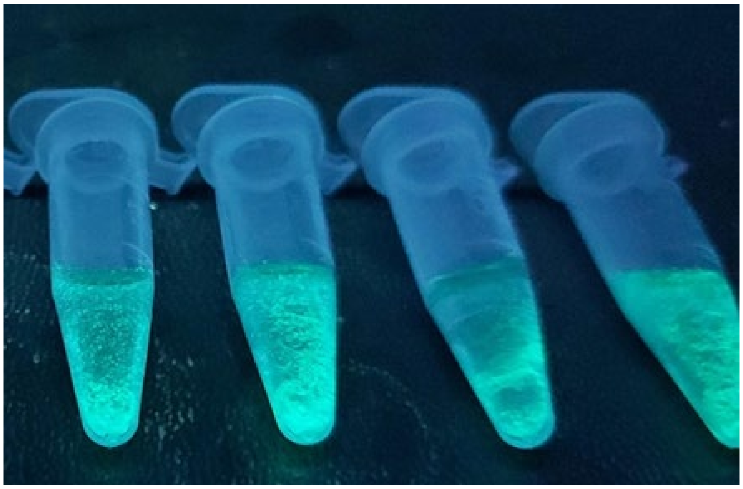

**Figure S29:** Discrete particles of gel formed when NaCl is added to a dilute (0.1-0.2 mM) sample of $[Au(6\text{-}tGH)_2]Cl$ . Photograph taken under illumination by an Hg lamp.

## 8. Au-S bond lengths for coordinated thione and thiolate ligands

### Thione (C=S)

In the crystal structure of **1**, [Au(6-tGH)$_2$]Cl, the carbon-sulfur distances are 1.701(12) and 1.726(13) Å.

On searching the CCDC for similar structures with linear coordination of sulfur to gold there are 99 hits.

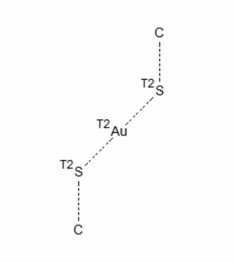


Of these 47 have been assigned to thione C=S [1-25]

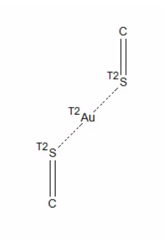


Of the 47 which have been assigned to the thione forms, the range of bond lengths is 1.653-1.779 Å with a mean bond length of 1.721 Å.

### Thiolate (C-S)

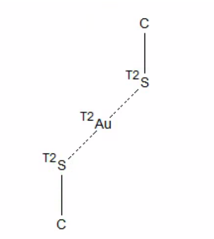


24 examples of single bond C-S that were not thiocyanates and could be clearly assigned as thiolate forms were found. [26-37] In this sample, the range of thiolate C-S bond lengths is 1.712-1.844 Å and the mean bond length is 1.772 Å.

### References for Au - thione and Au - thiolate bond lengths

(1) Yu, B.; Liu, Y.; Peng, X.; Hua, S.; Zhou, G.; Yan, K.; Liu, Y. Synthesis, characterization, and antitumor properties of Au(i)–thiourea complexes. *Metallomics* **2020**, *12*, 104-113.

(2) Piro, O. E.; Castellano, E. E.; Piatti, R. C.; Bolzán, A. E.; Arvia, A. J. Two thiourea-containing gold (I) complexes. *Acta Crystallographica Section C: Crystal Structure Communications* **2002**, *58*, m252-m255.

(3) Lobana, T. S.; Sharma, R.; Indoria, S.; Butcher, R. J. The influence of benzaldehyde-N-alkyl-thiosemicarbazones on the synthesis of gold (I) ionic complexes: Spectroscopy, ESI-mass, structures and variable H-bonded polymeric networks. *Polyhedron* **2015**, *91*, 89-97.

(4) Friedrichs, S.; Jones, P. G. Bis (imidazolidine-2-thione) gold (I) diiodoaurate (I). *Acta Crystallographica Section C: Crystal Structure Communications* **1999**, *55*, 1625-1627.

(5) Castiñeiras, A.; Fernández-Hermida, N.; Fernandez-Rodriguez, R.; García-Santos, I. Supramolecular Architecture in Gold (I) and Gold (III) 2-Pyridineformamide Thiosemicarbazone Complexes by Secondary Interactions: Synthesis, Structures, and Luminescent Properties. *Crystal growth & design* **2012**, *12*, 1432-1442.

(6) Hussain, M. S.; Isab, A. A. Bis (N-propyl-1, 3-imidazolidine-2-thione) gold (I) chloride: Crystal and molecular structure. *Transition Metal Chemistry* **1985**, *10*, 178-181.

(7) Jones, P. G.; GuY, J. J.; Sheldrick, G. Bis (ethylenethiourea) gold (I) chloride hydrate. *Acta Crystallographica Section B: Structural Crystallography and Crystal Chemistry* **1976**, *32*, 3321-3322.

(8) Cingi, M. B.; Bigoli, F.; Lanfranchi, M.; Leporati, E.; Pellinghelli, M. A.; Foglia, C. 4-Amino-3-methyl-1, 2, 4-Δ2-triazoline-5-thione: an example of thione-thiol tautomerism and stabilization of Cu (I) and Au (I) complexes. *Inorganica chimica acta* **1995**, *235*, 37-43.

(9) Hapka, M.; Dranka, M.; Orłowska, K.; Chałasiński, G.; Szczęśniak, M. M.; Zachara, J. Noncovalent interactions determine the conformation of aurophilic complexes with 2-mercapto-4-methyl-5-thiazoleacetic acid ligands. *Dalton Transactions* **2015**, *44*, 13641-13650.

(10) Patel, D. V.; Mihalcik, D. J.; Kreisel, K. A.; Yap, G. P.; Zakharov, L. N.; Kassel, W. S.; Rheingold, A. L.; Rabinovich, D. Tris (mercaptoimidazolyl) borate complexes of the coinage metals: syntheses and molecular structures of the first gold compounds and related copper and silver derivatives. *Dalton Transactions* **2005**, 2410-2416.

(11) Porter, L. C.; Fackler, J.; Costamagna, J.; Schmidt, R. Structure of bis (thiourea) gold (I) bromide,[Au {SC (NH2) 2} 2] Br. *Acta Crystallographica Section C: Crystal Structure Communications* **1992**, *48*, 1751-1754.

(12) Khanye, S. D.; Báthori, N. B.; Smith, G. S.; Chibale, K. Gold (i) derived thiosemicarbazone complexes with rare halogen–halogen interaction–reduction of [Au (damp-C 1, N) Cl 2]. *Dalton Transactions* **2010**, *39*, 2697-2700.

(13) Yan, K.; Lok, C.-N.; Bierla, K.; Che, C.-M. Gold (I) complex of N, N′-disubstituted cyclic thiourea with in vitro and in vivo anticancer properties—potent tight-binding inhibition of thioredoxin reductase. *Chemical communications* **2010**, *46*, 7691-7693.

(14) Castiñeiras, A.; Pedrido, R. Aurophilicity in gold (I) thiosemicarbazone clusters. *Dalton Transactions* **2012**, *41*, 1363-1372.

(15) Räisänen, M. T.; Runeberg, N.; Klinga, M.; Nieger, M.; Bolte, M.; Pyykkö, P.; Leskelä, M.; Repo, T. Coordination of pyridinethiols in gold (I) complexes. *Inorganic chemistry* **2007**, *46*, 9954-9960.

(16) Kolari, K.; Sahamies, J.; Kalenius, E.; Novikov, A. S.; Kukushkin, V. Y.; Haukka, M. Metallophilic interactions in polymeric group 11 thiols. *Solid State Sciences* **2016**, *60*, 92-98.

(17) Caddeo, F.; Fernández-Moreira, V.; Arca, M.; Laguna, A.; Lippolis, V.; Gimeno, M. C. Gold thione complexes. *Inorganics* **2014**, *2*, 424-432.

(18) Quillian, B.; Haddock, J. W. Imidazolidinethione Gold (I) and Copper (I) Halides: Synthesis, Characterization And Structure. *Structural Chemistry* **2015**, *1*, 5.

(19) Staples, R. J.; Fackler, J. P.; Costamagna, J. Bis (N, N'-dimethylthiourea-S) gold (I) Perchlorate and Bis (N, N'-diethylthiourea-S) gold (I) Perchlorate. *Acta Crystallographica Section C: Crystal Structure Communications* **1997**, *53*, 1555-1558.

(20) Patel, D. V.; Kreisel, K. A.; Yap, G. P.; Rabinovich, D. Gold (I) tris (mercaptoimidazolyl) borate chemistry: Synthesis and molecular structure of the first trinuclear TmR complex of a transition metal. *Inorganic Chemistry Communications* **2006**, *9*, 748-750.

(21) Usón, R.; Laguna, A.; Laguna, M.; Jiménez, J.; Gómez, M. P.; Sainz, A.; Jones, P. G. Gold complexes with heterocyclic thiones as ligands. X-Ray structure determination of [Au (C 5 H 5 NS) 2] ClO 4. *Journal of the Chemical Society, Dalton Transactions* **1990**, 3457-3463.

(22) Isaia, F.; Aragoni, M. C.; Arca, M.; Caltagirone, C.; Demartin, F.; Garau, A.; Lippolis, V. Gold oxidative dissolution by (thioamide)–I 2 adducts. *Dalton Transactions* **2013**, *42*, 492-498.

(23) Jones, P. G.; Friedrichs, S. Secondary interactions in gold (I) complexes with thione ligands. 5. Two ionic compounds of the form bis (thione) gold (I) bis (4-halobenzenesulfonyl) amide. *Acta Crystallographica Section C: Crystal Structure Communications* **2006**, *62*, m623-m627.

(24) Castineiras, A.; Dehnen, S.; Fuchs, A.; García-Santos, I.; Sevillano, P. Stabilization of gold (I) and gold (III) complexes by pyridil bis {3-hexamethylene-iminylthiosemicarbazone}: spectroscopic, structural and computational study. *Dalton Transactions* **2009**, 2731-2739.

(25) Strey, M.; Jones, P. G. Strukturen zweier Salze des Bis (thioharnstoff) gold (I)-Kations. *Zeitschrift für Naturforschung B* **2018**, *73*, 349-354.

(26) Seiji, W.; Takayuki, K.; Yasuchika, H.; Nobuko, K.; Masami, N.; Yasushi, K.; Shozo, Y. Blue Phosphorescence of the Novel Dinuclear Gold(I) Complex Bridged by 1,3-Benzenedithiolate in Solution. *Bulletin of the Chemical Society of Japan* **2004**, *77*, 531-536.

(27) Kenji, N.; Ryusuke, N.; Tomohiro, S. Synthesis and Crystal Structure of a Water-soluble Gold(I) Complex, {K3[Au(mba)2]}2 Formed by 2-Mercaptobenzoic Acid (H2mba), with Aurophilic Interaction in the Solid-State. *Chemistry Letters* **2000**, *29*, 274-275.

(28) J. LeBlanc, D.; W. Smith, R.; Wang, Z.; E. Howard-Lock, H.; late) Colin, J. L. L. Thiomalate complexes of gold(I): preparation, characterization and crystal structures of 1∶2 gold to thiomalate complexes*Journal of the Chemical Society, Dalton Transactions* **1997**, 3263-3268.

(29) Neils, T. L.; Biros, S. M.; Staples, R. J.; Wackerle, B. G.; Harrison, R. B. Three monomeric compounds containing the dipyrimidine-2-thiolategold(I) anion. *Polyhedron* **2019**, *157*, 474-478.

(30) Bates, P. A.; Waters, J. M. Structure of tetraphenylphosphonium bis(benzenethiolato)aurate(I), [P(C6H5)4][Au(C6H5S)2]. *Acta Crystallographica Section C* **1985**, *41*, 862-865.

(31) Schröter, I.; Strähle, J. Thiolatokomplexe des einwertigen Golds. Synthese und Struktur von [(2,4,6-iPr3C6H2S)Au]6 und (NH4)[(2,4,6-iPr3C6H2S)2Au]. *Chemische Berichte* **1991**, *124*, 2161-2164.

(32) LeBlanc, D. J.; Britten, J. F.; Wang, Z.; Howard-Lock, H. E.; Lock, C. J. L. Monoammonium Bis(d-penicillaminato-S)aurate(I) 3.667-Hydrate. *Acta Crystallographica Section C* **1997**, *53*, 1763-1765.

(33) Nöth, H.; Beck, W.; Burger, K. The Molecular Structure of Some Transition-Metal Complexes with 1, 2, 3, 4-Tetrazole-5-thiolate Anions. *European Journal of Inorganic Chemistry* **1998**, *1998*, 93-99.

(34) Bachman, R. E.; Bodolosky-Bettis, S. A.; Pyle, C. J.; Gray, M. A. Reversible oxidative addition and reductive elimination of fluorinated disulfides at gold (i) thiolate complexes: a new ligand exchange mechanism. *Journal of the American Chemical Society* **2008**, *130*, 14303-14310.

(35) Fujisawa, K.; Imai, S.; Moro-oka, Y. Two-coordinate gold (I) aliphatic thiolate complex: The Au-S distance is smaller than the Ag-S one. *Chemistry letters* **1998**, *27*, 167-168.

(36) Watase, S.; Kitamura, T.; Kanehisa, N.; Nakamoto, M.; Kai, Y.; Yanagida, S. Temperature-Dependent Solid-state Luminescence and Reversible Phase Transition of (n-Bu4N)[Au (SC6H3-3, 5-Me2) 2]. *Chemistry letters* **2003**, *32*, 1002-1003.

(37) Bates, P.; Waters, J. Structure of tetraphenylphosphonium bis (benzenethiolato) aurate (I),[P (C6H5) 4][Au (C6H5S) 2]. *Acta Crystallographica Section C: Crystal Structure Communications* **1985**, *41*, 862-865.
